# Linking Genetic Variation in Adaptive Plant Traits to Climate in Tetraploid and Octoploid Basin Wildrye [*Leymus cinereus* (Scribn. & Merr.) A. Love] in the Western U.S.

**DOI:** 10.1371/journal.pone.0148982

**Published:** 2016-02-16

**Authors:** R. C. Johnson, Ken Vance-Borland

**Affiliations:** 1 Agricultural Research Service, Plant Germplasm Research and Testing, United States Department of Agriculture, Pullman, WA, United States of America; 2 Conservation Planning Institute, Corvallis, OR, United States of America; National Cheng-Kung University, TAIWAN

## Abstract

Few studies have assessed how ploidy type within a species affects genetic variation among populations in relation to source climates. Basin wildrye (*Leymus cinereus* (Scribn. & Merr.) A. Love) is a large bunchgrass common in the intermountain Western U.S. found in both octoploid and tetraploid types. In common gardens at two sites over two years differences in both ploidy type and genetic variation within ploidy were observed in phenology, morphology, and production traits on 57 octoploid and 52 tetraploid basin wildrye from the intermountain Western U.S. (P<0.01). Octoploids had larger leaves, longer culms, and greater crown circumference than tetraploids but the numerical ranges of plant traits and their source climates overlapped between ploidy types. Still, among populations octoploids often had greater genetic variation for traits and occupied more diverse climates than tetraploids. Genetic variation for both ploidy types was linked to source climates in canonical correlation analysis, with the first two variates explaining 70% of the variation. Regression of those canonical variates with seed source climate variables produced models that explained 64% and 38% of the variation, respectively, and were used to map 15 seed zones covering 673258 km^2^. Utilization of these seed zones will help ensure restoration with adaptive seed sources for both ploidy types. The link between genetic traits and seed source climates suggests climate driven natural selection and adaptive evolution in basin wildrye. The more diverse climates occupied by octoploids and higher trait variation suggests a higher capacity for ecological differentiation than tetraploids in the intermountain Western U.S.

## Introduction

Western U.S. landscapes are challenged by the presence of frequent fires, exotic weeds, and changing climates that interact to limit ecosystem function [1, 2]. Over the last 20 years there has been an increasing emphasis on the use of local native plant germplasm in restoration to maintain local adaptation and promote ecological restoration [3]. Although genetically diverse native germplasm is increasingly desired, seed zones—geographic boundaries for genetically appropriate germplasm movement—are lacking or have not been implemented for key restoration species. By using common garden experiments based on a diverse sampling of populations across the landscape, genetic variation within a species can be assessed, and when linked to population source climate, it suggests climate driven adaptation though natural selection [4, 5].

Locally derived germplasm has often been shown to have an adaptive advantage [6, 7]. In a survey of 74 reciprocal transplant studies, Hereford [8] found local populations were more fit in 71% of the cases and differences between home site environments were positively associated with the magnitude of local adaptation. In a similar survey, Leimu and Fisher [9] reported the same result, and also found that local adaptation was much more common in larger than smaller populations. There are exceptions to the “local is best” paradigm [8–12], but in most studies, ecological relationships, essential for plant community and ecosystem function, are not considered. Moreover, smaller, less fecund plants may be better adapted to higher stress climates than larger, more fecund plants often associated lower stress climates [13, 14]. Longer term reciprocal transplant studies can more directly evaluate adaptation than the common garden approach but practical considerations usually limit the number of seed sources that can be included [5, 15]. Wang et al. [16] conducted landscape scale reciprocal transplant studies with lodgepole pine (*Pinus contorta* Dougl. ex Loud.). Nevertheless, including a larger number of populations from differing climates is usually more practical in common garden than reciprocal transplant studies [5, 15, 16]. Common gardens studies assessing genetic variation among climatically diverse source populations to develop and map seed zones are often referred to ‘genecology’ studies [13, 14, 17]. This may be the best current approach to guide restoration, maintain genetic diversity, and promote ecological relationships over large geographic areas.

Among the important restoration species on western U.S. rangelands is basin wildrye (*Leymus cinereus* (Scribn. & Merr.) A. Love). It is widely distributed, provides forage and cover for livestock and wildlife, and wind and water erosion control [18]. It is caespitose, self incompatible, wind pollinated, and found up to 2700 m above sea level in climates with precipitation varying from approximately 125 to more than 500 mm [18]. Basin wildrye is commonly found in the semi-arid to arid intermountain Western United States, a region from the Sierra Nevada and Cascade Mountain ranges in the west to the Rocky Mountains in the east. The region includes desert sage-shrub lands, temperate grasslands, and at higher elevations, montane coniferous forests.

Basin wildrye exists as a tetraploid (2n = 4x = 28) and an octoploid (2n = 8x = 56) type with the basic genomes designated as Ns and Xm [19, 20]. The Ns genome originated in the *Psathyrostachys*, Russian wildrye complex but the origin of the Xm genome is still uncertain [20, 21]. Both the octoploid and tetraploid basin wildrye are among the largest and most robust native grasses in North America. Consistent with its higher ploidy octoploid basin wildrye is generally larger than the tetraploid [22]. Still, the tetraploid cultivar ‘Trailhead’ was comparable in production to the octoploid cultivar ‘Magnar’ tested in Montana [23].

Since polyploids and their progenitors are usually reproductively isolated they can exist sympatrically [24], and this is the case for part of the basin wildrye range in the intermountain West [25]. Still, polyploidy can be a defining factor for ecological differentiation in an expanding range of environments [22, 26–29]. Differing ploidy has been associated with differences in phenology, morphology, and reproductive potential [22, 29, 30]. Polyploidy may also promote variation within populations perhaps as a reservoir of heterogeneity to enhance differentiation in new and a wider range of environments [22, 29]. With water deficits, higher ploidy has been associated with higher fitness [27] and favorable physiological responses such as enhanced photosynthesis and water relations [29, 31, 32]. Even with exceptions [33], higher ploidy has been associated with potential adaptations to water deficits and lower ploidy with more mesic or wetter environments [27, 31, 32].

Few studies have assessed genetic variation of adaptive traits among populations differing in ploidy in relation to source population climates [22], and we known of none that have modeled and mapped adaptive seed zones in a species differing in ploidy. Our specific objectives were to determine if 1) diverse ecotypes of basin wildrye varied genetically in adaptive traits between and within ploidy types, 2) genetic variation between and within ploidy was linked to source population climates, 3) ploidy affected the trait variation among and within populations, 4) the interaction between genetic variation and source climates could be mapped across the landscape to delineate seed transfer zones for both ploidy types.

## Materials and Methods

### Population sampling and garden establishment

Seeds from wild plants were collected at 110 source populations from the intermountain West in the spring of 2009. All collections were made on public, multiple use lands as permitted by Bureau of Land Management (BLM), U.S. Forest Service (USFS), and along roadsides where there were no restrictions on small seed collections of common, unthreatened plant species for research. The collections were funded by the BLM, USFS Great Basin Native Plant Project to improve the use of genetically appropriate seed sources for restoration. Approximately 10 inflorescences were collected separately from two individual plants spaced a least 5 m apart and from populations of at least 25 plants. Seeds derived from individual plants collected at a given location were maintained separately as half-sib families. Latitude, longitude, and elevation were recorded at each source population using geographic positioning instrumentation. For each collection source “annual” climate norms were extracted from ClimateWNA version 5.10 climate data rasters for the time period spanning 1981 to 2010 [34]. There were 23 annual climate variables designated including directly calculated means for temperature and precipitation, warmest and coldest months, summer precipitation, and heat to moisture indices. Additional derived variables included degree day indices, frost free days, precipitation as snow, 30 year minimum and maximum temperature extremes, evaporative demand indices, solar radiation, and relative humidity. As might be expected, correlation between climate variables was often strong; when that occurred repeatedly for a variable at r> ±0.90 it was considered redundant.

The 110 wild populations together with two cultivars were established at Central Ferry Research Farm, Central Ferry, WA (hereafter CF) and the Plant Germplasm Research Farm at Pullman, WA, (hereafter PU). The cultivars were the octoploid ‘Magnar’ (PI 469222) and the tetraploid ‘Trailhead’ (PI 478831) both supplied through the National Plant Germplasm System (http://www.ars-grin.gov/npgs/). According to National Plant Germplasm records Magnar was originally collected in 1939 in Saskatchewan, Canada, and developed by selecting vigorous plants over several generations at Aberdeen, ID by the Natural Resources Conservation Service. It was released as a cultivar in 1979 [18]. Trailhead was derived from a population collected near Roundup, MT in 1959 [23].

The Central Ferry farm (46.6698 N, -117.7557 W) is a lower elevation (209 m), relatively warm site in the Snake River Canyon; the PU location (46.7205 N, -117.1424 W) is a cooler, higher elevation (739 m) site representative of a steppe-prairie. The 30 year (1981–2010) mean annual temperature at CF was 12.1°C and 8.8°C at PU. The 30 year norm for precipitation at CF is 383 mm and for PU 455 mm.

In the winter of 2009 seeds from the 110 wild accessions and the 2 cultivars were germinated in boxes (13.3 cm long, 12.7 wide and 3.5 cm deep) containing water saturated vermiculate at room temperature (~20°C). Germinates were planted into 5 x 5 x 5 cm containers in flats holding 36 containers of Sunshine #5 plug mix (SunGro Horticulture, Bellevue, WA) and grown under greenhouse conditions. Seedlings were watered and fertilized as needed to promote optimal growth.

Seedlings were transplanted on 11 May 2010 at CF and on 7 Sept 2010 at PU after two weeks acclimation in a lath-house. The later planting at PU resulted from land procurement and preparation issues. The experimental design was a randomized complete block with six replications and 110 germplasm sources at both common garden sites. Plants were spaced 1.5 m apart in all directions. Within each block, wild collections from each source were represented by two families. Thus there were 6 individuals from each family for a total of 12 from each source population at each site. For cultivars, two plants from each source were included in each block also giving 12 individuals at each site. The experimental unit consisted of the two plants representing a family or cultivar. At each garden site there were 1344 plants. No fertilizer or irrigation was applied at either site and weeds were controlled by hand around each plant and with a small tractor cultivator in rows between plants. Plants were allowed to establish and grow through 2011 and data collection was completed in the spring of 2012 and 2013.

In 2012 and 2013 and at both sites, each plant was evaluated for phenology, production, and morphology traits defined in Table 1. The large number of plants and their large size, ranging up to 3.5 kg dry weight and 2.3 m tall, made it prohibitive to measure above ground dry weight on each plant at each garden site over years. However, random samples of dry weight, cut after seed maturity approximately 15 cm above ground level, correlated strongly with crown circumference (r = 0.81, n = 463), thus providing a surrogate for dry weight. After seed maturity each year remaining plants were mowed to approximately 15 cm and plant material removed from the plots.

**Table 1:** Plant traits measured on basin wildrye plants in common gardens at Central Ferry and Pullman, WA.

Trait	Trait description
**Phenology**	
Heading, day of the year (doy)	Day of year when a lead inflorescence emerged entirely from leaf sheaths
Blooming, doy	Day of year when a lead inflorescence had protruding anthers
Maturity, doy	Day of year when 50% of the seeds in a lead inflorescence were dehiscent and hard
**Morphology**	
Leaf area and weight	Average area and dry weight of four penultimate leaves from lead culms
Leaf ratio	Leaf length to width
Specific leaf weight, mg cm^-2^	Leaf mass to area
Culm length, cm	Distance from the plant base to bottom of the inflorescence on a lead culm
Head length, cm	Inflorescence length on a lead culm
**Production**	
Survival frequency	Coded 1 (survived) or 0 (died)
Head number	Counted per plant
Crown circumference, cm	Measured directly with a flexible tape

### Ploidy determinations

Relative DNA was measured on fresh leaf material from each source by flow cytometry similar to Culumber et al. [25] with a Partec CyFlow^®^ Ploidy Analyzer (Münster, Germany). We used the “DAPI” (4’,6-diamidino-2-phenylinde) Partec CyStain UV precise P extraction buffer and staining buffer recommended by the manufacturer. Leaf material was taken from a minimum of 4 different plants from each source, kept on ice, and tissue homogenized by chopping into fine particle with a razor blade for 30 to 60 seconds in extraction buffer, incubated for 1½ to 2 min, and then filtered through 30 μm nylon mesh. Stain buffer was added and the mixture incubated in dark for 1 min before analysis.

Before analyzing wild populations, the CyFlow Ploidy Analyzer was checked for DNA-DAPI quality control by running a sample tube of Partec Calibration Beads. Following this, samples of the cultivars Trailhead and Magnar with known ploidy [25] were run, and the gain (signal amplification) was adjusted to the known ploidy and the configuration script saved. This script was used to run subsequent samples. Sample speed was constant at 1.0 μl/sec with more than 1000 particles analyzed in most samples (see S1 Fig for peaks associated with Trailhead and Magnar as ploidy standards).

### Statistical analysis

Analysis of variance was completed on each plant trait in Table 1 using the mixed procedure (Proc Mixed) in SAS/STAT version 9.2 as described in Littell et al. [35]. Prior to analysis the distribution of each trait was examined using the univariate procedure (Proc Univariate) in SAS. Distributions of all traits except survival appeared normal to approximate normal with near linear normal probability plots. However, survival means did attain approximate normality.

Variation among garden site, ploidy, source population within ploidy, and year as a repeated measurement were treated as fixed effects. The set of interactions among garden site, ploidy, population within ploidy, and year were also fixed. Random effects were blocks nested within sites (PU or CF) and families within source populations as in St.Clair et al. [13] and Johnson et al. [14].

Separate analyses of variance were also completed to compare cultivars and wild populations as groups. For that, families within source were averaged and identified as cultivars or wild populations for each ploidy type. The difference between wild populations and cultivars groups was determined with years and the year by group interaction using the block within group as a random effect.

For wild populations of octoploids and tetraploids the phenotypic variance components were estimated using PROC MIXED with all experimental factors treated as random. Then plastic and genetic components for source populations were computed as outlined by Scheiner and Goodnight [36]. For each ploidy type the plastic variance components were derived from sites, years, associated interactions, and the block within sites variation. The genetic variance components were population source and family within population source. The plastic components were summed and divided by the total phenotypic variation to obtain percent plastic variation. Likewise, the genetic components were divided by the total phenotypic variation and percent calculated for the total genetic fraction. Confidence intervals for octoploid or tetraploid variance components were calculated at P = 0.05 and P = 0.01 on plant traits using the appropriate critical values of the Chi-square distribution as outlined by Snedecor and Cochran [37]. Non-overlapping confidence intervals were declared different at P<0.05 or P<0.01.

Canonical correlation (PROC CANCORR in SAS/STAT version 9.2) was used to assess the relationship between plant traits measured in common gardens and source population climate variables similar to Johnson et al. [14]. This resulted in canonical variates or linear combinations of traits and climate that maximized their correlation. Each set of variates for traits and climate represent independent dimensions in data, with the first variate having the highest correlation and the last the lowest [38].

Because there were 12 plant traits measured at each site and year, there was a potential for 48 trait combinations in the canonical correlation analysis, 12 for each of four year-site environmental combinations. Such a high number of potential traits complicates the identification of a small set of canonical variates explaining a high fraction of the variation suitable for modeling with climate variables. Pearson correlation analysis of traits between each year-site combination was completed to determine the magnitude and direction of interactions. If the correlation coefficient was highly significant (P<0.01), indicating there was reasonable correspondence between traits, data were averaged over sites or years environments.

For regression modeling, canonical variates were used as composite plant traits to model and map trait variation with seed source climate. Additionally, representative production, phenology, and morphology traits that correlated with canonical correlation scores were also regressed on climate variables and mapped. Regression modeling between plant traits and climate variables was completed using SAS PROC REG SAS/STAT version 9.2. The objective was to find models with the highest predictive value with the fewest number of model parameters [39]. Within PROC REG the R-squared option was used along with the Akaike information criterion (AIC) to minimize over parameterization [40]. For a given trait, climate variables from each source were initially included in the modeling process. The final model selected was the combination of climate variables that produced the highest R-square with the lowest AIC statistic. These models maximize prediction capacity with the coefficients and variables functioning as a set. Owing to correlations among variables in the regression, they do not necessarily identify the most important, independent climate variables for a given trait [40].

### Regression model mapping and seed zone development

Spatial mapping of plant traits and canonical variates predicted from regression models was completed over level III Omernik ecoregions [41] using the grid algebra function (raster calculator) of the ArcGIS 9.3 Spatial Analyst extension (ESRI, Redlands, CA). Raster layers comprised of the relevant climate variables were converted to trait values by multiplying each climatic variable by each associated regression coefficient in the model and summing the results. Using the regression model error term, the 95% confidence interval was calculated and used as the mapping contour distance. To avoid extrapolation beyond the data, mapping was confined to the range of observed traits and canonical scores.

## Results

### Analyses of variance between and within ploidy types

Ploidy analysis revealed 57 octoploid populations and 52 tetraploid populations collected across the intermountain West sampling region. Analyses of variance confirmed that ploidy had a major effect on plant traits, but also that source populations within ploidy varied extensively (Table 2). Ploidy was significant (P<0.01) for all traits except leaf ratio and often interacted with garden site. Source populations within ploidy were significant for all traits and interacted with site for all traits except maturity. Plasticity was also apparent. Year effects or the year x site interactions were significant for all traits except survival, which was largely determined during the first year of growth (data not shown). No trait had a significant year x population within ploidy interaction. The three way interactions for year x site x ploidy were significant for blooming, maturity, head number, and crown circumference, and year x site x population within ploidy was significant for blooming and maturity (P<0.01) (data not shown).

**Table 2:** Summary of analyses for 109 basin wildrye source populations growing in common gardens at Central Ferry and Pullman, WA in 2012 and 2013.

Trait	Mean	Ploidy	Population within ploidy	Site	Site x ploidy	Site x Population within ploidy
		^______________________________________________^F (P-values)^______________________________________________^				
**Phenology**						
Heading, doy^a^	154	62.6 (<.002)	4.0 (<.003)	2082 (<.001)	15.5 (<.004)	1.5 (<.005)
Blooming, doy	166	61.7 (<.001)	3.7 (<.001)	1901 (<.001)	12.5 (<.001)	2.1 (<.001)
Maturity, doy	217	29.3 (<.001)	3.2 (<.001)	13376 (<.001)	2.5 (0.112)	1.3 (0.013)
**Morphology**						
Leaf weight, g	0.304	472 (<.001)	10.3 (<.001)	16.8 (0.002)	18.9 (<.001)	2.4 (<.001)
Leaf ratio	31.6	0.16 (0.69)	1.8 (<.001)	0.21 (0.65)	0.12 (0.72)	0.94 (0.64)
Leaf area, cm^2^	28.1	359 (<.001)	11.0 (<.001)	21.7 (0.001)	25.9 (<.001)	2.7 (<.001)
Specific leaf wt., mg cm^-2^	10.7	108 (<.001)	2.3 (<.001)	3.9 (0.076)	2.9 (0.088)	1.5 (<.001)
Culm length, cm	139	150 (<.001)	2.8 (<.001)	20.2 (<.001)	1.3 (0.25)	1.5 (0.001)
Head length, cm	18.3	22.0 (<.001)	3.0 (<.001)	11.1 (0.007)	0.0 (0.95)	1.7 (<.001)
**Production**						
Survival frequency	0.75	41.4 (<.001)	2.0 (<.001)	46.3 (<.001)	14.2 (<.001)	2.6 (<.001)
Head number	116	64.0 (<.001)	2.2 (<.001)	0.7 (0.44)	19.1 (<.001)	1.9 (<.001)
Crown circumference, cm	85.4	272 (<.001)	6.0 (<.001)	7.7 (0.019)	71.2 (<.001)	3.1 (<.001)

^a^doy, day of year

Correlations between each of the four site-year combinations for each trait were positive and significant (P<0.01) in all cases. Thus, the interactions were always of magnitude rather than than direction with a general correspondence between results from the four different year-site combinations. Therefore, traits were averaged across common garden sites and years for Pearson linear correlation and canonical correlation analyses with climate variables.

### Ploidy mean comparisons

Although numerically small, differences between octoploid and tetraploid sources for heading and blooming were always significant for each site and year combination, with octoploids always developing later (Table 3). For maturity, differences associated with ploidy were observed only at PU in 2012, and at CF 2013. For morphology and production traits, octoploids were generally more robust, exhibiting greater leaf weight, specific leaf area, culm and head length, survival, and crown circumference (Table 3). Ploidy comparisons for leaf ratio, leaf area, and head number did not always differ for different year and site combinations.

**Table 3:** Mean comparisons between wild octoploid (n = 57) and tetraploid (n = 52) basin wildrye among years and common gardens sites at Central Ferry (CF) and Pullman (PU), WA.

			Phenology^b^			Morphology^c^						Production^d^		
Site	Year	Ploidy	Heading	Bloom	Maturity	Leaf weight	Leaf ratio	Leaf area	Specific leaf wt	Culm length	Head length	Surv.	Head number	Crown circum.
CF	2012	Octo	147.6**^a^	159.5**	207.3	0.368**	32.3**	32.6	11.3**	136.3**	18.7**	0.76**	114.8**	92**
		Tetra	146.5	157.2	207.3	0.248	30.0	23.3	10.6	120.5	17.6	0.59	73.1	62
PU	2012	Octo	166.3**	179.5**	227.8**	0.347**	32.1	30.5	11.3**	134.2**	17.6**	0.87**	54.1	58.3**
		Tetra	164.1	177.9	227.2	0.250	31.1	24	10.4	119.9	16.4	0.78	43.3	46.2
CF	2013	Octo	146.7**	157.3**	207.2**	0.409**	33.0**	36**	11.3**	151.1**	19.5**	0.76**	160.8**	115.1**
		Tetra	144.9	155.1	205.9	0.293	31.3	27.2	10.8	140.5	18.5	0.59	132.3	91.9
PU	2013	Octo	161**	170.4**	226	0.295**	32.6	28**	10.5**	164.5**	19.3**	0.87**	186.6**	117.3**
		Tetra	158.2	169.4	226	0.216	31.4	22.1	9.7	149.5	18.4	0.78	161.8	98.8
		Pooled se	0.333	0.345	0.157	0.0102	0.394	0.680	0.0002	1.27	0.238	0.0234	8.28	2.80

^a^,**within a garden site and year indicate a difference between ploidy for a given trait using the LSD (P<0.01)

^b^day of year

^c^leaf weight, g; leaf ratio, length to width; leaf area, cm^2^; specific leaf weight, mg cm^-2^; culm and head length, cm

^d^survival fraction; crown circumference, cm

Numerical values for traits and climate ranges overlapped between ploidy types such that the distribution of traits and climates were continuous rather than clustered into separate octoploid and tetraploid groups. For example, heading ranged from 150 to 163 for octoploids and 148 to 163 for tetraploids. Leaf area ranged from 19.7 to 46.3 for octoploids and 14.5 to 40.9 for tetraploids, head number from 84 to 199 for octoploids and 54 to 155 for tetraploids. Crown circumference had the same pattern. So even though octoploids had more heads and greater circumferences on average, many tetraploids had higher production than octoploids in common gardens.

The cold climate origins of the octoploid cultivar Magnar (Saskatchewan, Canada) and the tetraploid cultivar Trailhead (Roundup, Montana, USA) would suggest winter adaption, yet neither survived the first winter after transplanting at PU. We can only speculate why that occurred; perhaps poor acclimation conditions, population shift/drift or unfavorable interactions with biotic factors at PU. The cultivars also originated east of the Rocky Mountains rather than the intermountain West. Among all wild populations, however, there was only one source out of 110 that had no surviving plants at either site.

The cultivars did survive at CF, and as with wild collections, the octoploid Magnar was generally more robust than the tetraploid Trailhead. Compared to Trailhead, Magnar had higher leaf area (48.3 compared to 36.4 cm^2^), more heads (197 compared to 138), and greater circumference (154 compared to 108 cm) (P<0.0001). Wild octoploids averaged 138 heads, 104 cm circumference, and 34.5 cm^2^ leaf area, all substantially lower than Magnar. Likewise, wild tetraploids averaged 106 heads, 79 cm circumference and 26.4 cm^2^ leaf area, substantially lower averages than Trailhead. Thus, selection for plant production was apparently a high priority in cultivar development of both ploidy types.

### Variance components between ploidy types

The different environments associated with garden sites, years, and their interactions resulted in high plasticity values, especially for phenology (Table 4). Although plasticity was the largest component of total variation, it did not differ between ploidy for any trait.

**Table 4:** Comparisons of octoploid (n = 57) and tetraploid (n = 52) variance components for basin wildrye traits measured in common gardens at Central Ferry and Pullman, WA in 2011 and 2012.

Trait	Total plastic^a^		Total genetic^b^		Genetic among populations		Genetic families within populations	
	Octo	Tetra	Octo	Tetra	Octo	Tetra	Octo	Tetra
	--------------------------------------Percent of total variance--------------------------------------							
**Phenology**								
Heading	94.0	97.3	6.0	2.7	5.1	0.1	0.9	2.6
Blooming	96.2	99.9	3.8	1.7	3.2	0.1	0.5	1.5
Maturity	99.7	99.9	0.3	0.1	0.2	0.1	0.1	0.0
**Morphology**								
Leaf weight	65.2	76.3	34.8	23.7	32.1	18.1	2.7	5.7
Leaf ratio	93.1	96.4	6.9	3.6	3.6	2.4	3.3	1.2
Leaf area	58.9	70.3	41.1	29.7	38.3	22.1	2.7	7.6
Specific leaf weight	83.0	89.5	17.0	10.5	9.5	4.2	7.4	6.3
Culm length	85.6	84.8	14.4	15.2	8.6	7.6	5.8	7.6
Head length	70.3	77.6	29.7	22.4	15.6	14.7	14.0	7.8
**Production**								
Survival	87.1	86.0	12.9	14.0	3.0	8.5	9.8	5.4
Head number	91.2	94.5	8.8	5.5	5.3	0.9	3.5	4.6
Crown circumference	78.4	93.7	21.6	6.2	17.5	3.3	4.1	3.0
	----------------------------------------------Variance^c^-------------------------------------------------							
**Phenology**								
Heading	154.4	139.8	9.79*	3.83	8.39**	0.13	1.40	3.70
Blooming	167.9	180.9	6.57*	3.01	5.63**	0.23	0.94*	2.78
Maturity	199.9	201.9	0.51**	0.15	0.40*	0.14	0.11**	0.01
**Morphology**								
Leaf weight	0.0145	0.0077	0.0078*	0.0024	0.0072**	0.0018	0.0006	0.0006
Leaf ratio	49.3	48.5	3.6	1.8	1.9	1.2	1.8*	0.6
Leaf area	85.9	49.5	59.9*	20.9	55.9**	15.5	4.0	5.4
Specific leaf weight	1.72	1.67	0.35	0.20	0.20	0.08	0.15	0.12
Culm length	571.2	633.1	96.1	113.7	57.3	56.7	38.8	57.0
Head length	11.1	12.7	4.7	3.7	2.5	2.4	2.2	1.3
**Production**								
Survival	0.134	0.182	0.020	0.030	0.005**	0.019	0.015	0.012
Head number	8194.5	7326.8	792.3	427.5	475.4**	69.8	316.9	357.8
Crown circumference	1443.2	1209.5	397.2**	80.6	322.0**	42.1	75.2	38.4

^a^The sum of year, year x site, year x population, year x population x site, and block within site.

^b^The sum of population and families within populations.

^c^Variances between ploidy were declared significant when the confidence intervals at P<0.05 (*) and P<0.01 (**) did not overlap for a given trait and variance component.

Genetic variation was significantly higher for octoploids than tetraploids for all the phenology traits and for leaf weight, leaf area, and crown circumference (Table 4). When genetic variation was further partitioned, the among population component was significant for 8 of 12 traits and except for survival, octoploids traits were numerically higher in all cases. For families within populations, ploidy differed for blooming, maturity, and leaf ratio but neither octoploids or tetraploids were consistently higher.

### Linear correlation of traits and climate within ploidy

For both ploidy types significant correlation coefficients (P<0.05) indicated source populations from locations with higher continentality, lower precipitation, more extreme high temperatures, higher moisture deficits, and lower relative humidity tended to have earlier heading and blooming in the common gardens (S1 Table). For maturity the same was true for continentality and relative humidity.

Correlation of morphology and production traits were less consistent than phenology traits and more dependent on ploidy (S1 Table). For some traits significant correlation coefficients were in opposite directions and that was associated with the Columbia Plateau octoploids. As a group they had among the highest values of leaf weight, leaf area, head number, and crown circumference coupled with higher average temperatures and extreme minimum temperature. As a result, significant correlations of those traits with average temperature and extreme minimums were often positive in slope for octoploids and negative for tetraploids (S1 Table).

### Canonical correlation among adaptive traits

Canonical correlation using data from both ploidy types produced variates that established a clear link between source population plant traits and their home climates. Canonical correlations for variates 1 and 2 were 0.82 and 0.69, respectively. Both variates were significant (P<0.0001, S2 Table); variate 1 and 2 explained 49% and 21% of the variation, rescpectiviely, for a total or 70%. Thus the first two variates explained a high fraction of the total variation both between and within ploidy and were suitable as composite traits for regression modeling with climate variables.

### Regression models and mapping

**Fig 1:**
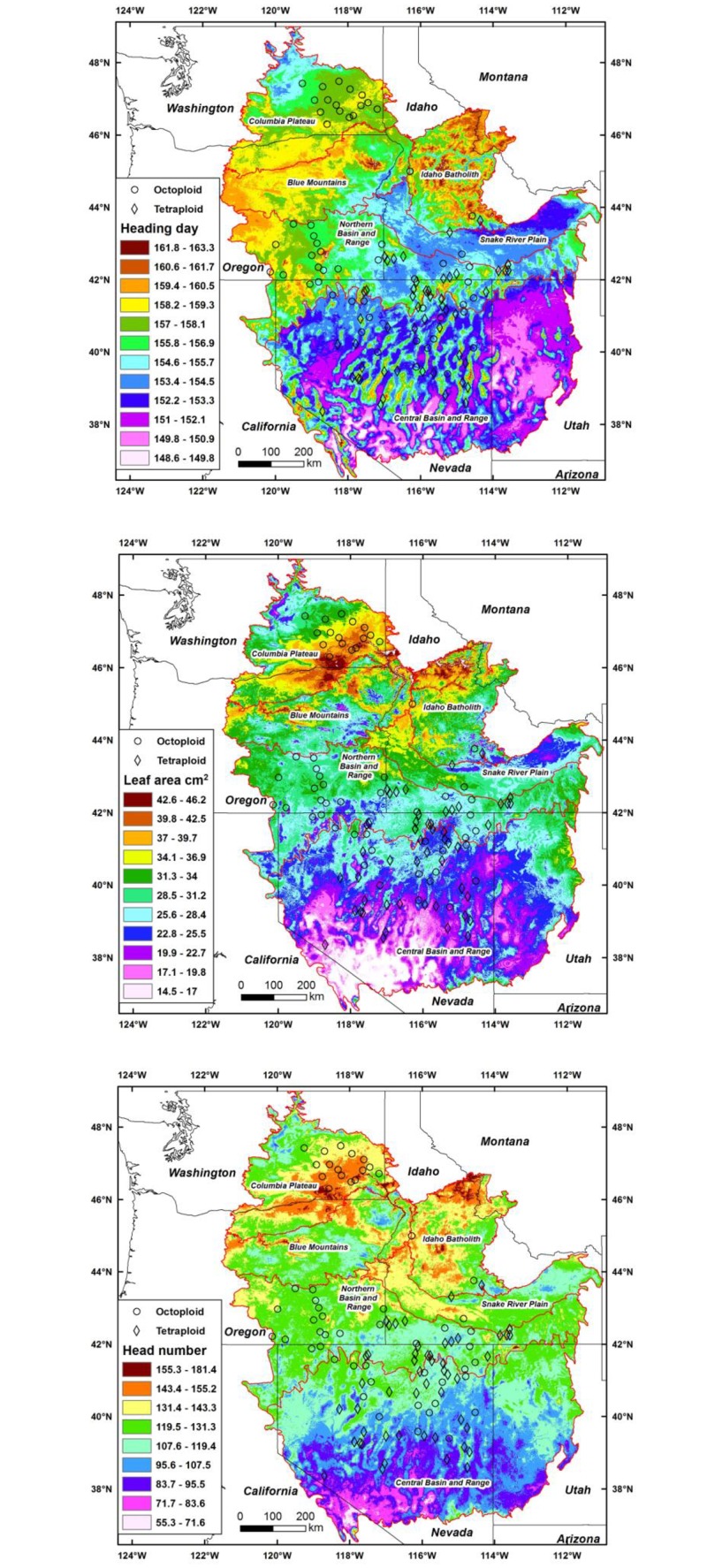
Maps of regression models for heading day, leaf area, and head number with climate variables for basin wildrye in the intermountain west. Omernik ecoregion boundaries are shown as red lines. The different colored areas were delimited with contours based on the ± P = 0.01 confidence interval from the regression model error term. Model predictions outside the range of trait values were not mapped (shown in white).

Regression models of genetic variation and source climates for a key phenology trait (heading day), morphology trait (leaf area), and a production trait (head number) were highly significant (P<0.0001) and explained 39% of the variation for heading, 47% for leaf area, and 29% for head number (S3 Table). Even with numerous exceptions, maps indicated later heading, more heads, and larger leaves in the northern areas dominated by octoploids compared to more southern regions (Fig 1). The region in the eastern Columbia Plateau, Blue Mountains, Idaho Batholith, and the western portion of the Northern Basin and Range was generally associated with somewhat higher precipitation, lower evaporative deficits, lower continentality, and higher relative humidity than areas where octoploids and tetraploids were sympatric, especially in the Northern and Central Basin and Range. The particularly high leaf area and head number typical of Columbia Plateau (Fig 1) was associated with higher average temperatures and higher extreme minimum temperatures

**Fig 2:**
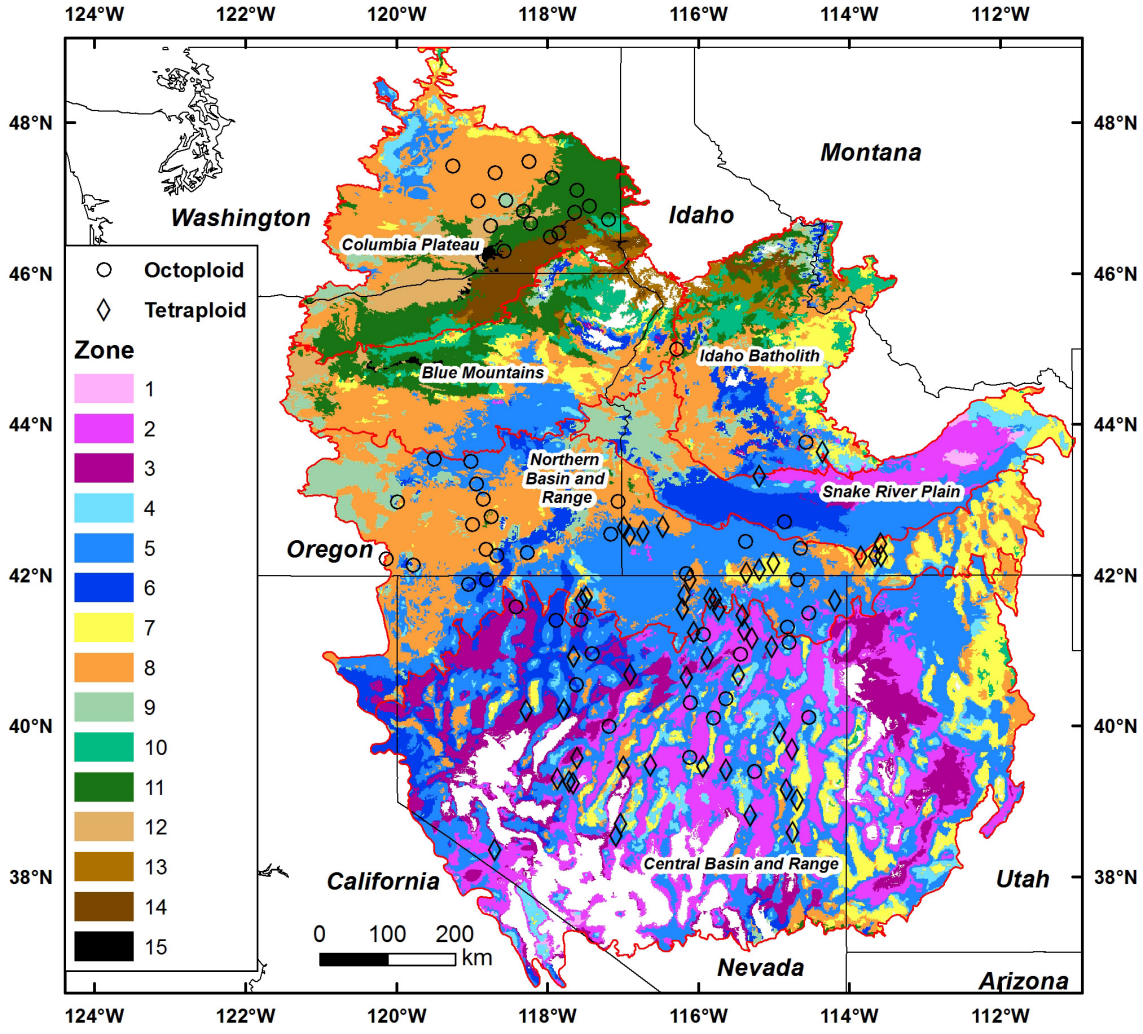
Fifteen proposed seed zones for basin wildrye based on regression models of canonical variates 1 and 2 with climate variables over intermountain west. The canonical variates were derived from canonical correlation of plant traits and climate variables. Ecoregion boundaries are shown as red lines. Model predictions outside the range of observed canonical scores were not mapped, and were shown in white.

Models for canonical variates 1 and 2 were highly significant (P<0.0001) and explained 64 and 38% of the variation, respectively (S3 Table). Given the distributions of heading day, leaf area, and head number we expected a general northern to southern distribution when canonical variates were modeled and overlaid, and that was the case (Fig 2). The overlay consisted of 15 zones, the product of 5 segments from the canonical variate 1 regression with climate model and 3 segments from canonical variate 2 regression with climate model. Greater weight was given to canonical variate 1 as it explained a higher portion of the variation (S3 Table).

The mapped area encompassed 673258 Km^2^ with three zones representing 58% of the mapped area (zone 2, 11.7%; zone 5, 26.5%; zone 8, 19.8%). Zone 1 in the eastern Snake River Plain and zone 15 in the Columbia Plateau were very minor with each occupying just 0.2% of the total area. The remaining zones represented from 1.1% to 8.2% of the area, totaling to 17% or 113304 Km^2^. Each of the three major zones contained both octoploid and tetraploid source populations; zone 2 with 53% tetraploid (19 total populations), zone 5 with 67% tetraploid (37 total populations), and zone 8 with 63% octoploid (22 total populations).

**Fig 3:**
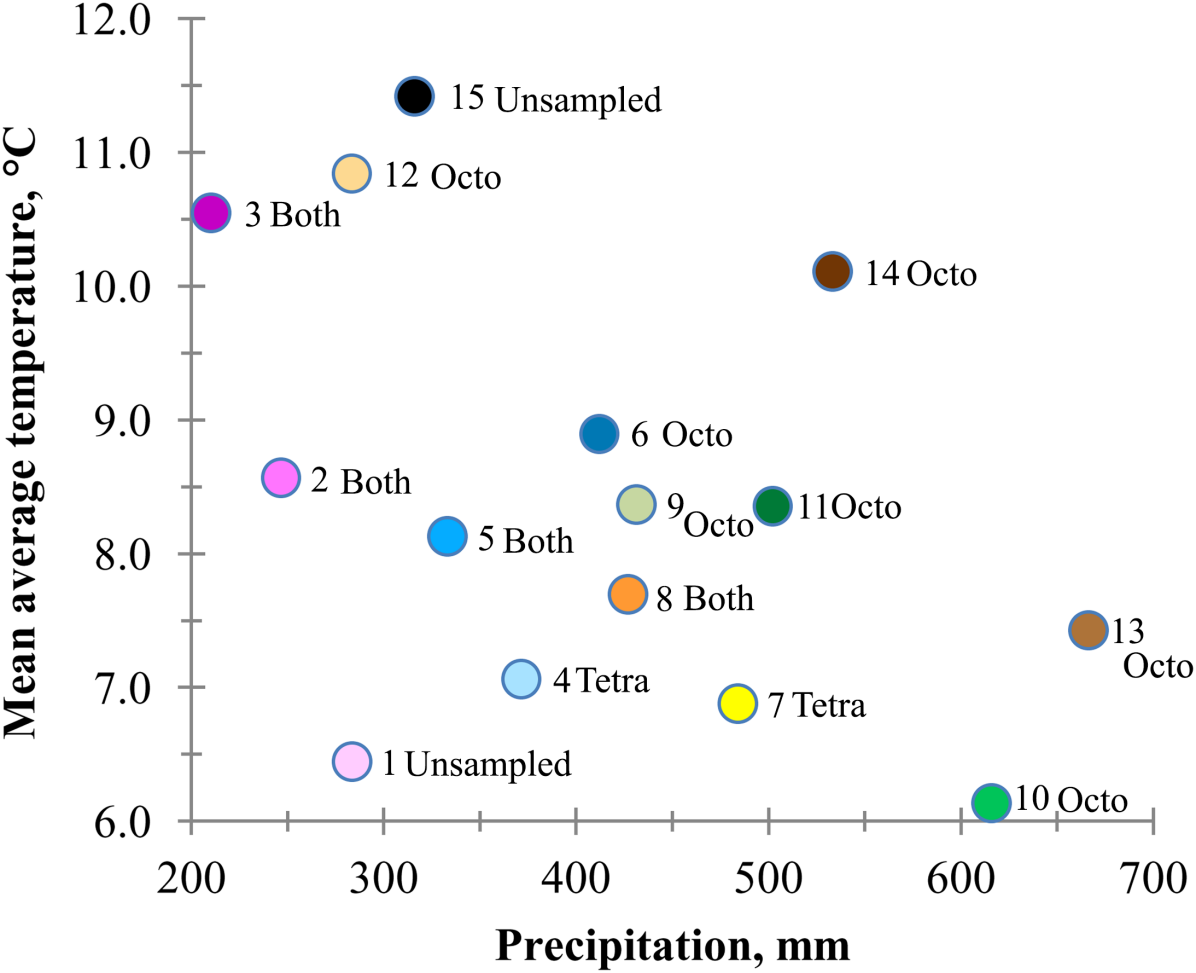
Relationship between average temperature and precipitation in basin wildrye seed zones (numbered 1 to 15) in the intermountain West. Populations within zones are designated as octoploid (octo), tetraploid (tetra), both, or unsampled.

Temperature and precipitation relationships among zones revealed a range from low precipitation and high temperature (high heat: moisture stress) to relatively high precipitation and low temperature (low heat: moisture stress) (Fig 3). In some cases the climatic transition from one zone to another was dramatic. The high stress zone 12 along the Columbia Gorge was adjacent to the considerably less stress prone zone 11 (Fig 2). Octoploids zones ranged from high stress (zones 3 and 12 high) to low stress (zone 10 and 13). But tetraploid zones were generally from areas never exceeding 500 mm precipitation and except for zone 3, were never above 8.6°C.

## Discussion

The widespread genetic variation among basin wildrye populations in the intermountain West (Table 2) was consistent with other grasses and forbs evaluated in common gardens [13, 14, 42, 43]. The same result has also been generally observed in other regions and species [9, 44, 45]. Culumber et al. [25] found extensive molecular variation among tetraploid and octoploid basin wildrye. Our study is however, the most comprehensive evaluation to date of adaptive quantitative traits and ploidy of diverse basin wildrye populations.

The capacity to respond to environmental changes through plasticity can contribute to overall fitness and sometimes be adaptive [46]. Plasticity has been defined in the strict sense as phenotypic changes of a single genotype with changes in environment [33, 40]. Here we have extended that to maternal half-sib families. Polyploidy has been posited to dampen plasticity associated with greater amplitude in fitness related traits. That was the case in the *Stellatia longipes* alloploid complex (MacDonald et al.) [47] but not for ploidy complexes of the grasses *Arrhenatherum elatius* [48], *Dactylis glomerata* [49], and *Brachypodium* [29]. In this study, plasticity resulting from the different garden sites, years and their interactions, was the largest component of total variation, but as with other grasses [29, 48, 49] plasticity did not vary significantly with ploidy (Table 4).

Genetic variation of octoploids, however, was generally higher than tetraploids (Table 4) and resulted mostly from variation among populations rather than variation within populations, a result similar to Petit and Thompson [48] for diploid and tetraploid *Arrhenatherum elatius*. The generally higher genetic variation of octoploids was associated with higher variation or diversity among octoploid home climates. For example, in the Columbia Plateau (Fig 2) seed zones ranged from the very warm and dry zone 12 to relatively cool and wet zone 10 (Fig 3). Higher ploidy has been associated with drought prone or high water deficit environments, and lower ploidy with more mesic or wetter environments [27, 31, 32]. Here, octoploids extended into dryer areas than did tetraploids, but were also found in relatively wet areas.

Higher genetic variation or diversity among octoploid populations represents a broadened gene pool that may facilitate natural selection through migration and gene flow [50]. Still, at the point of genome doubling from tetraploid to octoploid, genetic variation within or among populations is unknown, and differentiation associated with genetic drift and selection of alleles over time may dampen the direct effect of higher ploidy per se on ecological differentiation [22]. Nevertheless, even with the considerable overlap for ploidy in their climatic distributions, it appeared that basin wildrye octoploids had the capacity to adapt a wider range of climates than tetraploids.

We were able to unify the genetic and climate responses of both octoploid and tetraploid populations in a single seed zone map (Fig 2). The development of separate maps for each ploidy type was considered, but with the overlap of ploidy types in trait response and climate, that approach complicated rather than simplified the approach. Maps of both traits (Fig 1) and seed zones (Fig 2) illustrated the frequent overlap of populations with different ploidy types and their responses to climate. The three zones covering 58% of the mapped area contained both octoploid and tetraploid populations; zone 2 with 9 octoploids and 10 tetraploids, zone 5 with 14 octoploids and 23 tetraploids, and zone 8 with 14 octoploids and 8 tetraploids. Although seed zones represented both ploidy types on a landscape scale, differences in plant traits on a smaller climatic scale would also be expected [15]. Within zones 5 and 8 octoploids tended to originate from population locations that had somewhat higher temperature and lower precipitation than tetraploids. Still, with seed zones the prospect of large scale restoration with adapted, ecologically suited populations is substantially increased compared to the traditional practice using a limited number of cultivars.

The presence of overlapping ploidy types within zones makes choosing seed sources for restoration more challenging. Within a seed zone, ploidy determinations of collected populations is needed along with separate seed regeneration and storage. As information on ploidy and microclimate accumulates within zones, increasing informed decisions can be made by land managers.

As in similar studies [13, 14] we sought to balance seed zone size with the practical needs to apply seed zones to guide relatively large-scale restoration projects. Depending on the needs of land managers, there are cases when smaller scale restoration maybe needed. Using the same models (S3 Table), mapping detail could be increased by dividing the range of canonical variates into more sections. The disadvantage is that the number of seed zones over wide areas rapidly increases making large scale restoration more complex.

The seed zones are based on plant response from the seedling stage to maturity. As a result, germination, emergence and establishment was not directly considered. Therefore we encourage studies of plant establishment within the context of the seed zones. Given that genetic variation in growth and development of plant traits was linked to climate, perhaps differences in seed recruitment would also be linked. Espeland and Hammond [51] found Sandberg bluegrass performance at early growth stages was positively correlated with later stages.

The slopes of significant correlation coefficients (P<0.05) between traits and climate within ploidy offered insight into potential mechanisms of adaptation. For both octoploid and tetraploid populations, sources with earlier phenology in gardens were most typically from areas that were more continental, had less precipitation, had high moisture deficits, higher extreme temperatures, high solar radiation and lower relative humidity (S3 Table), representing increasingly stress prone climates. The trend toward earliness in climates with lower precipitation and high evapotranspiration potential is consistent with drought stress escape. Similar escape mechanisms were also observed with bluebunch wheatgrass (*Pseudoroegneria spicata* (Pursh) Á. Löve) [13] and Sandberg bluegrass [14] in the intermountain West.

Within octoploids, smaller and narrower leaves, fewer heads, and less plant circumference were associated with lower precipitation, higher moisture deficits, higher solar radiation and lower relative humidity (S3 Table); climatic factors that promote high evapotranspiration and drought stress. With water limiting, generally smaller plants would potentially use less water. With stress induced stomatal closure both smaller and narrower leaves would help reduce and dissipate increased sensible heat [52] resulting from reduced transpiration cooling [53]. These attributes, helping to maintain more favorable water relations and temperature conditions in the presence of stress, are consistent with stress avoidance mechanisms [54].

Leaf area for tetraploid sources trended smaller in climates with higher mean temperatures, higher summer heat: moisture indices, and higher minimum extreme temperatures. Together with solar radiation, the association of smaller leaves with higher stress climates fit the pattern observed in Sandberg bluegrass [14] and is also consistent with stress avoidance mechanisms [54].

Thus studies completed on bluebunch wheatgrass [13], Sandberg bluegrass [14] and now basin wildrye in the intermountain West indicate some general adaptive response patterns In environments with higher temperature or drought stress potential, escape is promoted though earlier phenology. In the presence of stress, avoidance mechanisms; that is, those that occur to maintain more favorable temperature conditions or water relations [54], are promoted with smaller and more narrow leaves and less above ground plant production. These generalities do not discount or exclude the existence of variants or stress tolerance mechanisms; that is, the ability to maintain plant metabolic function when plant tissue directly experiences lower water potential or unfavorable temperatures [29, 32, 54]. Still, escape and avoidance mechanism appear fundamental to general adaptation of these species in the intermountain West. Given these attributes for adaptation to diverse climates, seed zones are likely more suitable for restoration then the traditional approach of using few cultivars usually selected for high production [13].

Using correlation and regression analysis, this study provides evidence for climate driven natural selection and adaptation associated with both ploidy types. It would be useful to examine adaptation more directly through long term reciprocal transplant studies. This would be facilitated by selecting populations of both ploidy types from different seed zones and evaluating them within ‘home’ and ‘away’ zones. Also of interest is the ecological range and comparative genetic diversity of Basin wildrye populations in the Great Plains east of the Rocky Mountains. For basin wildrye in the intermountain West, octoploid and tetraploid populations often occupied the same seed zones, but octoploids had generally higher genetic variation and were found in more diverse climates than tetraploids. As a result, they appeared to have a capacity to adapt to and an expanded range of climates than tetraploids.

## Supporting Information

S1 Data Data for *Leymus cinereus* populations originating in the intermountain West, USA, taken in common gardens at Central Ferry and Pullman WA, 2012 and 2013. (PDF)

S1 Fig Output from the Partec CyFlow^®^ Ploidy Analyzer (Münster, Germany) used to determine relative DNA content of tetraploid and octoploid basin wildrye [*Leymus cinereus* (Scribn. & Merr.) A. Love]. The tetraploid cultivar Trailhead (peak 1) and the octoploid cultivar Magnar (peak 2) were used as standards to distinguish ploidy in 110 wild collections from the intermountain Western USA.
(TIF)

S1 Table Pearson correlation coefficients between climate variables and plant traits for octoploid (n = 57) and tetraploid (n = 52) basin wildrye source locations. Traits were measured in common gardens at Central Ferry and Pullman WA, in 2012 and 2013.
(DOCX)

S2 Table Canonical correlation summary for the first four variates relating plant traits from common gardens and source climates for basin wildrye. (DOCX)

S3 Table Regression models between traits and climates for octoploid (n = 57) and tetraploid (n = 52) source populations of basin wildrye in the intermountain West, U.S.A. (DOCX)
