# Correction: Linking Genetic Variation in Adaptive Plant Traits to Climate in Tetraploid and Octoploid Basin Wildrye [*Leymus cinereus* (Scribn. & Merr.) A. Love] in the Western U.S

**DOI:** 10.1371/journal.pone.0156921

**Published:** 2016-05-31

**Authors:** R. C. Johnson, Ken Vance-Borland

There is an error in Table 3. In the column for leaf ratio, the octoploid and tetraploid values were reversed, so when significant, the tetraploids rather than the octoploids had the greater leaf ratio values. The written descriptions of the results and discussion are not affected. Please see the corrected Table 3 here.

**Table 3:** Mean comparisons between wild octoploid (n = 57) and tetraploid (n = 52) basin wildrye among years and common gardens sites at Central Ferry (CF) and Pullman (PU), WA.

			Phenology^b^			Morphology^c^						Production^d^		
Site	Year	Ploidy	Heading	Bloom	Maturity	Leaf weight	Leaf ratio	Leaf area	Specific leaf wt	Culm length	Head length	Surv.	Head number	Crown circum.
CF	2012	Octo	147.6**^a^	159.5**	207.3	0.368**	30.0	32.6	11.3**	136.3**	18.7**	0.76**	114.8**	92**
		Tetra	146.5	157.2	207.3	0.248	32.3**	23.3	10.6	120.5	17.6	0.59	73.1	62
PU	2012	Octo	166.3**	179.5**	227.8**	0.347**	31.1	30.5	11.3**	134.2**	17.6**	0.87**	54.1	58.3**
		Tetra	164.1	177.9	227.2	0.250	32.1	24	10.4	119.9	16.4	0.78	43.3	46.2
CF	2013	Octo	146.7**	157.3**	207.2**	0.409**	31.3	36**	11.3**	151.1**	19.5**	0.76**	160.8**	115.1**
		Tetra	144.9	155.1	205.9	0.293	33.0**	27.2	10.8	140.5	18.5	0.59	132.3	91.9
PU	2013	Octo	161**	170.4**	226	0.295**	31.4	28**	10.5**	164.5**	19.3**	0.87**	186.6**	117.3**
		Tetra	158.2	169.4	226	0.216	32.6	22.1	9.7	149.5	18.4	0.78	161.8	98.8
		Pooled se	0.333	0.345	0.157	0.0102	0.394	0.680	0.0002	1.27	0.238	0.0234	8.28	2.80

^a,^**within a garden site and year indicate a difference between ploidy for a given trait using the LSD (P<0.01)

^b^day of year

^c^leaf weight, g; leaf ratio, length to width; leaf area, cm^2^; specific leaf weight, mg cm^-2^; culm and head length, cm

^d^survival fraction; crown circumference, cm
