# Supplementary material for: Linking Genetic Variation in Adaptive Plant Traits to Climate in Tetraploid and Octoploid Basin Wildrye [Leymus cinereus (Scribn. & Merr.) A. Love] in the Western U.S
Source: PLoS One. 2016 Feb 16;11(2):e0148982. doi: 10.1371/journal.pone.0148982 (PMC4755535; doi:10.1371/journal.pone.0148982)
Supplement: S1 Data — (PDF) [file pone.0148982.s001.pdf]

S1 Data. Data for Leymus cinereus populations originating in the intermountain West, USA taken in common gardens at Central Ferry and Pullman WA, 2012 and 2013

Survivial freqency; heading and blooming are "day of year"; leaf ratio is length over width; culm length, cm

Population	Ploidy	n	SeedZone	Survival	Heading Day	LeafRatio	BloomDay	CulmLength
915	Octoploid	48	12	0.79	156	26.77	166.1	145.1
997	Octoploid	48	14	0.92	156.1	25.38	167.4	157
1027	Octoploid	48	14	0.83	156.4	24.03	166.5	159
1115	Octoploid	48	12	0.92	158.4	25.58	169.5	141.4
1191	Octoploid	48	14	0.92	158	21.68	169	155.6
1247	Octoploid	48	8	0.79	162.3	33.37	172.2	150.6
1319	Octoploid	48	8	0.92	152.3	29.19	164.3	137.4
1401	Octoploid	48	9	0.75	162.3	29.1	172.3	164.6
1519	Octoploid	48	8	0.67	159.6	26.86	171.3	162.7
1611	Octoploid	48	11	0.92	153.4	26.74	162.7	144.4
1696	Octoploid	48	11	0.83	154.6	26.43	165.2	140.7
1909	Octoploid	48	11	0.88	159.1	27.82	168.3	145.8
1946	Octoploid	48	11	0.92	158.4	29.64	168.6	148.9
2126	Octoploid	48	11	0.91	161.4	30.29	170	159.8
2149	Octoploid	48	8	0.96	158.7	26.88	168.7	149.4
2467	Octoploid	48	10	0.92	159.3	23.72	170.4	157.1
2963	Octoploid	48	6	0.67	153.5	32.34	165.1	150.3
3258	Octoploid	48	5	0.79	155.3	30.04	167.3	155.2
3911	Octoploid	48	8	0.96	157.2	24.63	168.1	150.3
3970	Octoploid	48	5	0.92	157.7	29.96	170.1	165.8
4124	Octoploid	48	3	0.83	153.5	35.05	166.4	147
4137	Octoploid	48	5	0.79	160.9	31.75	170.3	152.7
4144	Octoploid	48	6	0.83	151.7	31.82	164.3	141.8
4167	Octoploid	48	8	0.88	157	27.22	168.6	149.7
4177	Octoploid	48	5	0.96	152.9	27.63	164.3	145.4
4222	Octoploid	48	5	0.79	156.7	32.2	167.4	146.8
4229	Octoploid	48	5	0.88	158.7	24.48	168.9	160.1
4255	Octoploid	48	5	0.92	153	31.18	165.1	154.3
4321	Octoploid	48	6	0.79	157.3	32.84	169.3	144.3
4350	Octoploid	48	8	0.67	155.3	30.51	168.6	143.4
4373	Octoploid	48	6	0.67	152.3	34.15	163.8	147.3
4409	Octoploid	48	8	0.67	163.3	34.41	173.2	153.9
4419	Octoploid	48	5	0.88	156.5	22.1	167.8	141.1
4429	Octoploid	48	5	0.75	155.4	32.3	167.2	153.4
4498	Tetraploid	48	3	0.83	153.1	33.91	164.5	152.7
4537	Octoploid	48	3	0.79	153.2	39.03	165.2	148.1
4554	Octoploid	48	8	0.38	156.2	31.55	169.1	146.1
4580	Octoploid	48	5	0.92	154	35.47	163.9	149.8
4583	Octoploid	48	8	0.92	153.2	30.42	165.9	143.3
4728	Tetraploid	48	8	0.63	156.1	33.22	167.6	125.2

4751	Octoploid	48	6	0.67	152.3	34.87	162.8	141
4783	Tetraploid	48	8	0.71	157.2	33.16	168.9	135.9
4874	Tetraploid	48	2	0.63	149.5	30.56	161.1	131.9
4875	Octoploid	48	6	0.92	155.6	31.77	167.4	152.8
4970	Octoploid	48	8	0.5	162.8	34.65	174	129
5003	Octoploid	48	2	0.79	152.7	33.42	164.4	149.6
5059	Tetraploid	48	5	0.71	154.3	35.23	164.9	138.4
5095	Tetraploid	48	5	0.73	153.9	34.29	165.5	133.2
5125	Octoploid	48	5	0.83	153	33.65	165.1	152.2
5203	Tetraploid	48	7	0.83	154.8	36.83	164.3	135
5213	Tetraploid	48	8	0.71	152.4	29.64	164.6	127.1
5236	Octoploid	48	2	0.67	153.4	37.18	163.2	146.8
5269	Tetraploid	48	2	0.79	153.5	33.93	165.7	126.1
5272	Tetraploid	48	5	0.79	153.1	31.63	164.4	136.2
5325	Tetraploid	48	3	0.79	154.8	34.95	165.6	132.4
5335	Octoploid	48	2	1	149.9	35.39	161.8	139.3
5420	Tetraploid	48	8	0.79	154.8	30.46	167.4	145.2
5433	Octoploid	48	2	0.88	155.5	33.57	168.4	135.4
5449	Tetraploid	48	5	0.33	155.1	33.26	168.1	119.9
5466	Tetraploid	48	2	0.58	155.3	32.7	168.4	131.4
5482	Tetraploid	48	4	0.67	152.9	32.17	164.1	133.8
5489	Octoploid	48	8	0.71	161	27.6	172	134.5
5515	Tetraploid	48	5	0.88	152.6	30.38	164.2	131.6
5561	Tetraploid	48	5	0.79	154.8	30.58	167	137.2
5562	Tetraploid	48	5	0.63	153.1	28.1	163.7	130.7
5591	Tetraploid	48	5	0.81	153.1	33.29	164.5	128.8
5604	Tetraploid	48	5	0.92	154.2	33.23	164.8	146.7
5682	Tetraploid	48	5	0.79	152.4	28.39	163.8	143.5
5791	Tetraploid	48	5	0.5	153.5	30.73	166.1	103.2
5810	Octoploid	48	2	0.88	151.8	34.7	164.1	130.5
5832	Octoploid	48	5	0.83	153.1	39.99	164.5	135.8
5833	Tetraploid	48	7	0.67	155.9	30.14	167.1	113
5906	Tetraploid	48	8	0.38	156.3	48.97	167	126.9
5918	Tetraploid	48	7	0.68	153.2	30.65	165.2	125.2
5919	Octoploid	48	8	0.6	156.5	26.44	168.1	134.9
6020	Octoploid	48	2	0.92	155	38.09	167	146.8
6050	Tetraploid	48	4	0.75	155.7	31.66	167.5	128.7
6053	Octoploid	48	5	0.83	151.3	38.49	163	140.9
6089	Octoploid	48	2	0.88	152.5	35.38	164.1	145.5
6093	Tetraploid	48	5	0.88	154.8	32.42	164.6	134.6
6112	Octoploid	48	2	0.64	153.2	36.73	166	137.6
6119	Tetraploid	48	8	0.79	155.3	29.43	166.8	130.3
6125	Octoploid	48	5	0.71	151.7	36.91	163.2	120.9
6129	Tetraploid	48	2	0.79	152.9	28.51	164.3	141
6142	Tetraploid	48	5	0.79	155.1	29.73	167.1	131.6
6155	Tetraploid	48	2	0.83	153.6	32.66	166	131.9
6188	Tetraploid	48	2	0.88	154.3	27.91	165.8	147

6224	Tetraploid	48	5	0.71	155.6	32.25	168.4	143.3
6247	Tetraploid	48	5	0.58	154.9	29.36	167.9	134
6263	Tetraploid	48	2	0.58	148.6	32.41	162.2	138.8
6289	Octoploid	48	2	0.92	152.9	33.71	164.6	133.2
6293	Tetraploid	48	2	0.71	155.9	30.59	167.3	135.8
6440	Tetraploid	48	2	0.75	152	38.54	163.1	122.9
6611	Tetraploid	48	2	0.79	152.6	30.92	165.3	131.8
6650	Tetraploid	48	8	0.58	154	32.12	166.5	119
6673	Tetraploid	48	5	0.96	153.7	27.09	164.7	167.8
6706	Tetraploid	48	5	0.67	154.2	31.31	167.2	131.2
6713	Tetraploid	48	7	0.58	156.7	27.35	169	141.4
6808	Tetraploid	48	4	0.67	156.6	31.89	167.1	133.7
6827	Tetraploid	48	5	0.83	151.7	30.45	163.4	139
6880	Tetraploid	48	7	0.79	153.6	29.02	166.5	136
6969	Tetraploid	48	5	0.46	158.9	35.1	170.3	135.3
7035	Octoploid	48	8	0.79	161.8	26.01	170.3	158.2
7057	Tetraploid	48	5	0.88	154.1	33.11	164	129.8
7136	Tetraploid	48	8	0.54	157.3	39.54	168.8	113.8
7162	Tetraploid	48	5	0.58	154.5	37.75	166.9	125.6
7208	Tetraploid	48	7	0.75	153.6	29.37	165.6	127.7
7808	Tetraploid	48	5	0.42	157.2	35.01	169.3	137.6
7930	Tetraploid	48	5	0.33	162.8	36.87	174.5	131.5

S1 Data. Data for Leymus cinereus populations originating in the intermountain West, USA taken in common gardens at Central Ferry and Pullman WA, 2012 and 2013

Head length, cm; maturity is "day of year"; crown circumference, cm; leaf area, cm^2; leaf weight, g						
Population	HeadLength	HeadNumber	MaturityDay	CrownCircum	AveLeafArea	AveLeafWt
915	16	127.6	217.9	98.6	33	0.3928
997	20.9	173.8	218.1	133.1	42.6	0.4454
1027	20.1	155.9	219.8	126.2	44.1	0.4744
1115	20.6	120	217.4	104.1	32.4	0.3718
1191	20.1	166.7	218.2	127.2	46.3	0.4738
1247	19.1	129.2	221.1	100.5	32.6	0.3407
1319	16.3	136.6	216.9	117.7	33.9	0.3608
1401	21.5	177.8	216.8	132.1	42	0.4799
1519	20.2	140.1	220.3	100.9	39.4	0.4022
1611	19.6	162.9	215.8	113.4	34.9	0.3916
1696	23.4	180.9	215.1	113.5	44.1	0.5299
1909	19	167.1	217.1	118.1	35.5	0.395
1946	21.3	134.8	216.3	106.9	37.1	0.4772
2126	21.7	159.7	218	130.8	44.8	0.4962
2149	19.8	199.1	217.4	124.1	41.2	0.4836
2467	20.6	132	218.3	118.6	44.7	0.5427
2963	18.2	125.9	216.2	94.4	31.6	0.3466
3258	21	131.4	218.3	93.5	34.3	0.3581
3911	18.6	160.1	217.7	115.4	39.5	0.4345
3970	19.5	98.9	218.2	88.1	34.9	0.3951
4124	16.7	102.1	215.7	72.9	28.1	0.3173
4137	20.8	153.3	219	104	32.9	0.3876
4144	17.4	108.8	216.7	72.3	22.7	0.223
4167	19.3	117.7	216.3	92.2	34.3	0.4088
4177	18.6	161.2	216.9	105.5	34.2	0.3749
4222	22.1	170.2	219.9	105.7	32.4	0.3522
4229	20.5	170.9	219.9	131.4	45.7	0.4801
4255	21.2	126.6	216.8	95.4	37.4	0.4288
4321	17.8	103.6	218.5	69.9	25.1	0.2977
4350	18.9	111.9	218.7	90.6	30.8	0.3591
4373	18.2	99.9	216.6	75.3	23.6	0.2812
4409	21.9	123.4	220.3	97	36.3	0.4146
4419	17.8	133.9	218.3	105.1	38.2	0.3984
4429	20.2	114.9	217.6	85.2	27.7	0.3268
4498	15.8	101.6	217.6	69.8	21.6	0.221
4537	18.9	101.2	218.3	69.2	26.2	0.2937
4554	18	83.6	218.6	77.1	30.1	0.3565
4580	19.4	126.6	216.8	88.3	30	0.3414
4583	18.4	140.7	217	88.7	29.6	0.3328
4728	19.5	104	218.6	84.2	28.4	0.3126

4751	18.2	131.3	216.8	89.2	23.4	0.2419
4783	17.5	105.9	218.4	83.4	26.3	0.2717
4874	17.9	117.5	215.3	76.1	28.4	0.2812
4875	19.6	145.7	216.7	91.8	31.5	0.3615
4970	17.4	147.5	219.9	87.3	26	0.2815
5003	17.2	136.7	217.6	82.5	22.7	0.2521
5059	20.3	110.3	217.2	77.7	29.1	0.3237
5095	16.8	96.2	218.7	75.9	18	0.2047
5125	17.7	123.8	216.9	84.9	29.3	0.3494
5203	20.4	108.9	217.1	87.6	31.1	0.3545
5213	16.2	103.8	217.2	71.6	23.6	0.2397
5236	15.8	101.1	215.5	67.9	22.4	0.2608
5269	13.7	86.6	217.9	80.4	21.2	0.2151
5272	23.4	114.1	216.2	73.8	28.8	0.3068
5325	19.1	126.7	218.9	75.6	21.6	0.2172
5335	15.3	103.1	215.8	78.3	21.2	0.2259
5420	18.8	103.8	218.6	74	32.4	0.3406
5433	15.1	94.1	217.5	72.8	24.7	0.2891
5449	13.5	75.8	220.6	63.3	15.2	0.1577
5466	17.5	123.5	219.7	75	22.6	0.2318
5482	18.1	113.8	216.6	76.6	28.7	0.2886
5489	16.7	103.8	221.1	78	23	0.2493
5515	17.7	118	216.2	76.7	26.6	0.2871
5561	18.3	107.3	218.8	74.9	25.8	0.256
5562	16.5	95.5	214.7	72.4	26.2	0.2693
5591	18.8	86.9	216.6	80.5	24	0.2359
5604	19.9	142	216.4	83.8	29.5	0.3102
5682	16.3	98.2	216.6	82.8	27.5	0.278
5791	14	91.5	219.8	56.8	18.1	0.1958
5810	15.4	93.6	217.1	71.3	20.8	0.2119
5832	16.9	114.2	217.7	83.8	20.3	0.2105
5833	17.8	65	214.9	63.5	25.3	0.28
5906	17.5	54.5	219.8	52.6	14.5	0.1569
5918	17.3	90.2	217	73.3	27.5	0.2981
5919	17.5	119.8	219.1	83.4	30.6	0.3291
6020	15.6	115.1	216.3	85	21.5	0.2443
6050	17	91.9	219.3	59.8	22.4	0.2302
6053	18.2	104.3	216.8	72	22.5	0.2406
6089	18.3	113	216.1	81.9	26.3	0.287
6093	18.5	144.1	216.9	76.2	26	0.2524
6112	17.6	110.1	218.3	70.2	19.7	0.2245
6119	19.9	107.3	215.5	83.8	26.6	0.266
6125	16.9	90	217	73.4	20.1	0.2182
6129	17.6	130.7	218.2	83	22	0.2241
6142	17.1	110.8	219.1	74.9	24.9	0.2411
6155	20.1	69.8	218	58.5	25.2	0.2635
6188	17.8	119.1	216.5	79.4	27.5	0.2668

6224	19.2	106.8	219.1	69.1	23.3	0.2246
6247	18.4	120.9	219	81.4	24.8	0.2502
6263	17	88.3	216.8	67.4	21.7	0.2227
6289	16.8	118	216.8	85.8	24.2	0.2576
6293	18.5	101.6	217.5	75.3	22.8	0.2338
6440	15.6	106.3	218	66.2	17.6	0.1723
6611	17.6	108.9	218.9	76.2	25.8	0.278
6650	16	88.6	217.6	66.3	23	0.2329
6673	20.9	155	217.4	108.2	40.9	0.4277
6706	17.5	113.4	219.3	76.4	22.1	0.2199
6713	18.6	108.1	220.8	78.2	26.1	0.2781
6808	19.2	88.4	218.9	67.9	21.8	0.2153
6827	17.5	130.8	216.3	82.8	20.6	0.2145
6880	18.9	103.4	219.1	79.5	23.4	0.2376
6969	15.8	106.3	222.2	69.9	17.4	0.1819
7035	23.2	165.1	218.3	128.3	44.2	0.4678
7057	18.4	91.9	218.1	70.4	24.4	0.2491
7136	14.5	98.6	221.8	75.7	18.1	0.1842
7162	14.5	83.3	218.9	65	16.9	0.1824
7208	18.7	101.5	216	77.7	28	0.2926
7808	17.3	137.3	222.6	73.1	23.2	0.2449
7930	16.5	67.9	221.5	66.3	19	0.199

S1 Data. Data for Leymus cinereus populations originating in the intermountain West, USA taken in common gardens at Central Ferry and Pullman WA, 2012 and 2013

	Specific leaf weight, g cm-2	First three canonical correlation scores						Mean ave temp (MAP)
Population		CanCorr1	CanCorr2	CanCorr3	Latitude	Longitude	Elevation	MAT
915	0.0116	1.25623	0.64961	0.1816	46.66434	-118.227	279	11.5
997	0.0103	1.55775	0.82249	0.64797	46.48526	-117.961	304	11.6
1027	0.0107	1.88756	0.17405	0.81205	46.29575	-118.581	313	11.4
1115	0.0114	1.39397	-0.83209	-0.37232	46.63162	-118.755	340	10.8
1191	0.01	1.845	0.21376	1.25997	46.53655	-117.855	363	11.4
1247	0.0104	0.97492	-0.68607	-0.51184	46.9613	-118.921	380	10.3
1319	0.0105	1.11135	0.74498	1.44432	47.42506	-119.258	402	10.1
1401	0.0114	2.17196	1.6784	-0.7491	46.96931	-118.558	427	10.1
1519	0.0103	0.49752	0.64683	-0.42481	47.33294	-118.698	463	9.6
1611	0.0112	0.86844	0.37572	0.95296	46.83036	-118.327	491	10.2
1696	0.012	2.10497	0.69361	0.26277	46.81092	-117.642	517	10.2
1909	0.011	1.56957	0.28419	0.03877	47.10358	-117.617	582	9.5
1946	0.0127	2.02662	0.55351	-0.32135	47.27052	-117.944	593	9.1
2126	0.011	2.45471	-0.09374	1.07497	46.89556	-117.45	648	9.3
2149	0.0117	1.93747	1.66693	-0.51574	47.48803	-118.252	655	8.4
2467	0.0121	2.9152	0.43381	0.06694	46.71218	-117.191	752	8.8
2963	0.0109	-0.05447	1.39735	0.57071	42.70683	-114.861	903	10.9
3258	0.0102	0.03197	0.29953	0.1957	42.8205	-116.162	993	10
3911	0.011	1.33639	0.87848	0.46828	44.99504	-116.293	1192	6.6
3970	0.0111	0.20799	0.94335	0.35101	42.54473	-117.168	1210	9.1
4124	0.0112	-0.97291	1.82918	0.82262	41.58105	-118.418	1257	9.6
4137	0.0117	1.06512	0.38742	-1.34807	43.50621	-119.02	1261	7.6
4144	0.01	-1.4665	0.42808	0.45284	41.40252	-117.891	1263	9.9
4167	0.0118	0.80735	0.73682	0.06165	43.0084	-118.855	1270	8.5
4177	0.0109	0.2721	1.21764	0.53116	43.20874	-118.948	1273	8
4222	0.0109	0.38711	0.26438	-1.30858	43.5406	-119.507	1287	7.5
4229	0.0106	1.84246	0.45738	0.60954	42.44897	-115.376	1289	9.3
4255	0.0114	0.2837	1.01532	0.98305	42.26353	-118.675	1297	9.8
4321	0.0118	-0.67665	0.8035	-1.09077	41.94057	-118.808	1317	9.5
4350	0.0115	0.56149	0.88051	-1.05116	42.96885	-119.992	1326	8.1
4373	0.0118	-1.06381	1.15932	-0.26215	40.96235	-117.409	1333	10.2
4409	0.0114	1.45109	-0.54853	-0.97641	42.97638	-117.064	1344	8.4
4419	0.0105	1.21204	-0.38899	0.91105	42.44775	-115.38	1347	9.2
4429	0.0118	-0.47082	1.08254	-0.93007	42.29634	-118.269	1350	9.2
4498	0.0102	-1.61251	0.89247	0.50187	40.68443	-116.902	1371	10
4537	0.011	-1.15189	0.76218	0.33417	41.4069	-117.563	1383	9.3
4554	0.0118	0.39872	0.87958	-1.12221	42.13251	-119.781	1388	9.3
4580	0.0113	-0.38488	0.56573	1.02022	42.35683	-114.653	1396	8.9
4583	0.0111	-0.3883	1.45231	-0.18232	42.67014	-118.995	1397	8.6
4728	0.0111	0.65627	-1.25017	-0.2907	42.41321	-113.582	1441	8.2

4751	0.0103	-0.65816	0.32224	0.14519	40.54926	-117.621	1448	10.1
4783	0.0105	-0.01636	-0.36308	-0.15284	42.24634	-113.844	1458	8.5
4874	0.0099	-0.73378	-0.16431	1.59261	40.64572	-116.159	1486	8.9
4875	0.0113	-0.22405	1.65624	-0.2748	41.87702	-119.048	1486	8.4
4970	0.0108	0.74619	0.1751	-2.54694	42.34539	-118.815	1515	8.7
5003	0.011	-1.32933	1.82996	-0.79004	39.99098	-117.186	1525	9.3
5059	0.0111	-0.2252	-0.4917	0.56045	42.63046	-116.987	1542	7.5
5095	0.011	-0.60916	-0.29078	-0.94724	40.20538	-118.278	1553	11.2
5125	0.0116	-0.45022	2.04326	0.0358	41.93911	-114.689	1562	7.8
5203	0.0112	0.60855	-1.20073	1.40218	42.25129	-113.575	1586	8
5213	0.0101	-0.47715	-0.54733	0.36436	42.02581	-115.364	1589	7.8
5236	0.0116	-1.44655	1.36141	0.73047	40.95	-115.445	1596	7.1
5269	0.0101	-0.09798	-0.42024	0.7776	39.29715	-117.864	1606	9.5
5272	0.0105	-0.4687	-1.47807	0.4893	42.5728	-116.73	1607	6.8
5325	0.0101	-0.75991	-0.95428	-0.53967	39.57454	-117.605	1623	9.1
5335	0.0107	-1.47461	1.02182	1.45303	40.30487	-116.108	1626	8
5420	0.0105	-0.39538	0.02311	0.6083	41.93718	-116.108	1652	7.4
5433	0.0113	-0.26754	0.87874	-0.00805	41.49982	-114.543	1656	7.3
5449	0.0104	-0.27482	-0.61359	-2.01536	40.2183	-117.781	1661	10.7
5466	0.0102	-0.46398	0.14771	-1.72179	41.16452	-115.291	1666	6.9
5482	0.01	-0.37908	-0.52744	1.1804	42.07432	-115.189	1671	7
5489	0.0111	0.22413	-1.06736	-1.99025	42.2186	-120.142	1673	6.9
5515	0.0107	-0.49774	-0.12193	0.76644	43.31722	-115.188	1681	6
5561	0.0099	-0.49389	-0.79422	0.01657	41.55272	-116.205	1695	7.6
5562	0.0102	-0.19983	-0.87947	1.45437	41.55272	-116.205	1695	7.6
5591	0.0099	-0.18527	-1.79675	1.3107	42.53262	-116.909	1704	6.8
5604	0.0104	-0.76043	0.37291	0.79275	43.63465	-114.35	1708	5.2
5682	0.0101	-0.42742	-0.1236	1.43966	41.73492	-116.185	1732	7.3
5791	0.0108	-0.18245	-1.32569	-1.27626	41.66096	-114.191	1765	7.2
5810	0.0102	-0.99755	-0.10141	0.92828	40.10527	-115.803	1771	7.5
5832	0.0104	-0.91323	0.35004	0.28664	40.36031	-115.643	1778	7.4
5833	0.0111	0.43517	-2.31921	1.11949	42.16466	-115.005	1778	6.9
5906	0.0106	-0.94863	-1.47765	-0.51317	40.92169	-117.652	1800	8.2
5918	0.0105	0.33042	-1.09306	0.83226	42.25095	-113.66	1804	7.1
5919	0.0106	0.66571	-0.48182	-0.61285	42.7792	-118.752	1804	6.5
6020	0.0113	-1.15527	2.08592	-0.01918	39.58405	-116.12	1835	7.9
6050	0.0103	-0.6703	-1.26112	-0.28054	41.70462	-117.489	1844	7.4
6053	0.0107	-1.47483	0.51061	0.76958	41.30827	-114.822	1845	7.1
6089	0.0108	-0.88251	0.79589	0.9321	40.11176	-114.541	1856	8.1
6093	0.0096	-0.73947	-0.80205	0.81246	40.9095	-115.879	1857	8.1
6112	0.0114	-1.4248	1.22718	-1.42572	41.21286	-115.939	1863	5.9
6119	0.01	0.07717	-1.47653	0.68038	42.63964	-116.467	1865	5.7
6125	0.0108	-0.51456	-0.84765	0.54407	41.1118	-114.802	1867	7.3
6129	0.0101	-0.75982	-0.01036	-0.67808	39.41676	-115.632	1868	8.2
6142	0.0096	-0.25993	-1.10458	0.01609	41.69162	-115.843	1872	5.5
6155	0.0104	-0.73211	-1.89642	0.78234	39.69083	-114.753	1876	8.3
6188	0.0097	-0.85448	-0.03475	0.91054	41.26999	-115.396	1886	6.7

6224	0.0096	-0.95026	-0.49644	-0.80729	41.68206	-115.776	1897	5
6247	0.0101	-0.19629	-0.20124	-1.35729	41.24463	-116.056	1904	6.3
6263	0.0102	-1.37242	0.60037	0.23978	39.47524	-116.631	1909	7.5
6289	0.0105	-0.40618	0.15256	0.66516	39.39587	-115.256	1917	7.8
6293	0.0103	-0.41247	-0.95865	-0.36927	41.46998	-115.414	1918	6.1
6440	0.0094	-0.72066	-1.04338	0.54383	39.24141	-117.658	1963	8.3
6611	0.0108	-0.47144	-0.12176	-0.19822	38.81628	-115.308	2015	7.6
6650	0.01	-0.05889	-1.17153	0.18663	41.04601	-115.026	2027	6.8
6673	0.0104	-0.01315	1.28886	1.47912	39.15904	-114.828	2034	7.3
6706	0.01	-0.50618	-0.36092	-1.08868	41.53851	-115.729	2044	5.5
6713	0.0106	0.03103	-0.56885	-1.75596	40.67441	-115.465	2046	6.2
6808	0.01	-0.39715	-2.22157	-0.24532	39.90978	-114.921	2075	7.6
6827	0.0104	-1.12326	0.59861	-0.3352	38.54139	-117.086	2081	8.6
6880	0.0101	-0.39055	-0.88845	-0.67473	38.58942	-114.747	2097	8.1
6969	0.0103	-0.31466	-0.49068	-2.66903	38.70159	-117.032	2124	8
7035	0.0105	2.25768	-0.93099	0.47798	43.76025	-114.574	2145	2.9
7057	0.0104	-0.53013	-2.04322	1.27179	39.02327	-114.689	2151	7
7136	0.0102	0.36352	-1.37737	-1.28475	39.45643	-116.996	2175	8
7162	0.0107	-0.78289	-0.01961	-0.78671	39.26502	-117.709	2183	7.5
7208	0.0103	0.10807	-1.09923	0.87536	39.46	-115.94	2197	7.4
7808	0.0105	-0.49285	0.53593	-2.82518	41.67096	-117.547	2380	4.3
7930	0.0106	0.27396	-1.38881	-2.38223	38.35808	-118.7	2417	6

S1 Data. Data for Leymus cinereus populations originating in the intermountain West, USA taken in common gardens at Central Ferry and Pullman WA, 2012 and 2013

	Continentality (TD), mean annual precip (MAP), summer heat: moisture index (SHM), extreme minimum temp (EMT), extreme maximum temp (EXT), climatic moisture deficit (CMD), mean annual solar radiation (MAR), mean annual relative humidity (RH)							
Population	TD	MAP	SHM	EMT	EXT	CMD	MAR	RH
915	22.6	283	328.2	-21.4	41.6	757	15.1	61
997	21.9	375	251	-20.5	41.3	700	15	62
1027	22.5	324	272.8	-21.4	41	725	15.8	61
1115	23.1	237	364.6	-23.5	41	786	15.7	59
1191	21.9	382	242.2	-21.1	41.2	687	15	61
1247	23.5	230	353.8	-25.6	40.7	767	15.6	59
1319	24	226	354.3	-26.5	40.7	769	14.8	59
1401	23.1	248	327.8	-25.1	40.8	766	15.3	58
1519	23.3	268	316.7	-26.8	40.5	745	15.3	58
1611	22.8	302	279.9	-24.7	40.8	730	15.4	59
1696	21.6	425	203	-23.3	40.6	645	15	60
1909	21.5	423	177	-24.4	40.4	617	15.1	60
1946	22.5	389	209.8	-26.8	40.2	658	15	58
2126	21	477	164.9	-24.8	39.7	579	15.4	61
2149	22.7	335	214.7	-27.9	39.3	648	14.4	59
2467	20.6	534	136.7	-25.2	39.1	508	16	63
2963	24.9	236	388.9	-28.2	43	930	17.2	51
3258	24	224	302.6	-28.7	42.5	854	17.4	53
3911	24	592	110.3	-35.5	40.2	562	17.4	50
3970	23.3	272	255.5	-30.1	41.4	796	17.2	52
4124	24.4	167	516.8	-31.9	42.3	981	17.8	47
4137	22.5	245	270.7	-32.7	39.5	770	19	50
4144	24.2	234	320.4	-30.6	42.4	914	17.8	48
4167	22.2	253	218.1	-30.9	40.2	784	18.4	50
4177	22	240	236	-31.5	39.5	774	18.9	51
4222	21.4	222	250.1	-32.2	39.7	772	18.8	50
4229	23.6	221	280.8	-29.9	41.4	821	17.4	53
4255	23.1	177	390.6	-28.6	40.5	875	17.9	53
4321	23.1	193	361.8	-29.9	41.2	906	17.8	50
4350	20.5	188	233.2	-29.9	40.1	825	17.8	49
4373	25	212	356.6	-30.8	41.8	932	18.5	50
4409	23.2	339	213.4	-31.7	40.8	733	17.5	51
4419	23.4	226	265.4	-30	41.2	804	17.4	53
4429	22.1	202	318.9	-29.3	39.8	832	17.7	53
4498	24.6	227	302.7	-31.8	42.6	971	18	46
4537	23.7	267	352.6	-31.5	41.9	877	17.9	49
4554	21.5	260	283.7	-27.9	40.8	803	17.7	53
4580	23.4	259	214.2	-30.6	40.4	759	17.3	53
4583	21.6	249	205.9	-29.7	40.2	772	17.4	52
4728	23	342	158.6	-32.2	40.3	698	16.9	51

4751	24.5	229	352.4	-31	41.9	925	18	49
4783	23.6	306	158.6	-31.6	40.5	708	17.1	52
4874	25.3	210	282.6	-35.4	42.1	922	17.8	45
4875	22	187	313.4	-30.8	40.2	846	17.9	50
4970	21.8	311	212.5	-28.1	39.5	694	17.8	57
5003	24.1	207	323.9	-33.2	41.6	931	18.1	48
5059	23.1	349	215.8	-33.3	40	697	17.5	51
5095	24.2	280	275.7	-26.2	40.6	811	18.8	59
5125	23.8	221	197.1	-35	40.5	824	16.9	46
5203	24.8	324	154.2	-33.3	39.9	663	17.1	54
5213	21.8	309	170.6	-33.4	41	732	17.2	47
5236	24.3	281	221.1	-38.7	42.8	850	17.9	41
5269	23.3	231	288.9	-33.2	40.9	943	18.7	45
5272	23.5	361	198.2	-35.6	40.4	692	17.5	49
5325	23.6	223	298	-33.7	41	937	18.3	45
5335	24.1	267	233.9	-36	41.6	866	17.9	44
5420	22.7	341	182.6	-34.2	40.3	711	17.4	48
5433	25	244	217.4	-37.1	41.3	834	17.3	44
5449	23.8	255	312.1	-27.4	41	845	19.7	56
5466	24.5	273	223.5	-38.4	41	827	17.9	42
5482	21.8	301	160.8	-35.5	40.3	715	17.3	46
5489	19.8	414	161.6	-30.4	38	611	18.3	54
5515	24.4	445	195.4	-36.7	39.3	642	19	50
5561	22.9	365	174.7	-33	41	693	18.4	51
5562	22.9	365	174.7	-33	41	693	18.4	51
5591	22.9	368	181.1	-33.2	39.2	633	17.6	54
5604	24.6	452	138.7	-40.3	39.1	616	17.9	46
5682	22.6	406	174.1	-33.7	40.6	671	17.8	50
5791	24.7	278	179.2	-35.7	40.4	741	17.6	49
5810	24	286	209.6	-36.2	40.9	792	18.4	46
5832	23.9	344	175.5	-35.5	40.7	730	18	47
5833	21.6	325	151.4	-34.2	39.3	647	17.7	50
5906	23	378	197.1	-29.1	38.2	611	17.9	61
5918	23.8	532	134.2	-31.5	37.9	497	18	61
5919	20.2	624	122.6	-29.6	36.8	461	17.8	60
6020	23.7	257	203.3	-35.1	40.2	819	18.6	47
6050	21.5	512	147.8	-29.4	38.1	523	18	61
6053	24.2	304	181.8	-35.6	40.2	725	17.7	49
6089	24.5	223	243.2	-35.5	40.3	842	18.2	47
6093	24.2	326	216.7	-31.9	39.9	690	17.6	56
6112	23	287	204.1	-37.4	39.5	733	18.2	47
6119	22.6	471	123.9	-35.7	38.9	557	18.2	51
6125	24.2	302	191.4	-35	40.2	733	18.2	50
6129	23.8	286	212.7	-35.2	40.2	810	18.5	47
6142	23.4	363	164.2	-38.7	39.9	658	18.3	46
6155	24.2	216	227.3	-35.4	40.6	853	18	46
6188	23.8	281	213.7	-35.7	39.8	730	18.2	50

6224	23.5	346	163.7	-40.3	39.9	674	17.8	43
6247	22.6	297	210.5	-34.9	39.2	691	18.2	51
6263	22.9	236	226.8	-36.6	39.8	848	18.9	44
6289	23.7	238	205.6	-36.7	39.9	844	18.4	44
6293	23.4	256	218.1	-36.3	39.3	719	17.5	50
6440	22.9	218	325.7	-34.2	40	874	20	47
6611	23.3	314	163.3	-36.3	39.2	787	20	46
6650	23	513	143.1	-34.2	39.1	582	17.8	53
6673	23.8	275	181.2	-37.1	39.3	795	18.6	45
6706	22.8	378	157.2	-36.6	38.7	604	18.1	51
6713	22.3	511	128.2	-34.2	38.1	505	17.8	55
6808	23	322	189.8	-33.8	39.2	710	21.3	51
6827	22.3	235	239	-31.3	38.8	813	19.7	52
6880	23.5	343	158.2	-34	39	730	19	50
6969	22.1	274	222.1	-31.7	37.9	749	18.5	52
7035	22.9	720	81	-42.4	36	422	18.6	46
7057	23.7	318	148.4	-37	38.7	729	19.3	46
7136	22.3	364	190.3	-30.5	38.2	658	19	55
7162	21.7	295	229.2	-32.4	38.2	751	20.3	50
7208	22.6	378	162.2	-32.2	37.9	614	18.6	56
7808	21.2	750	117.8	-32.7	34.2	381	18.6	65
7930	19.3	324	199.6	-34.1	36	676	20.9	50
