# Supplementary figures and images for: Linking Genetic Variation in Adaptive Plant Traits to Climate in Tetraploid and Octoploid Basin Wildrye [Leymus cinereus (Scribn. & Merr.) A. Love] in the Western U.S
Source: PLoS One. 2016 Feb 16;11(2):e0148982. doi: 10.1371/journal.pone.0148982 (PMC4755535; doi:10.1371/journal.pone.0148982)

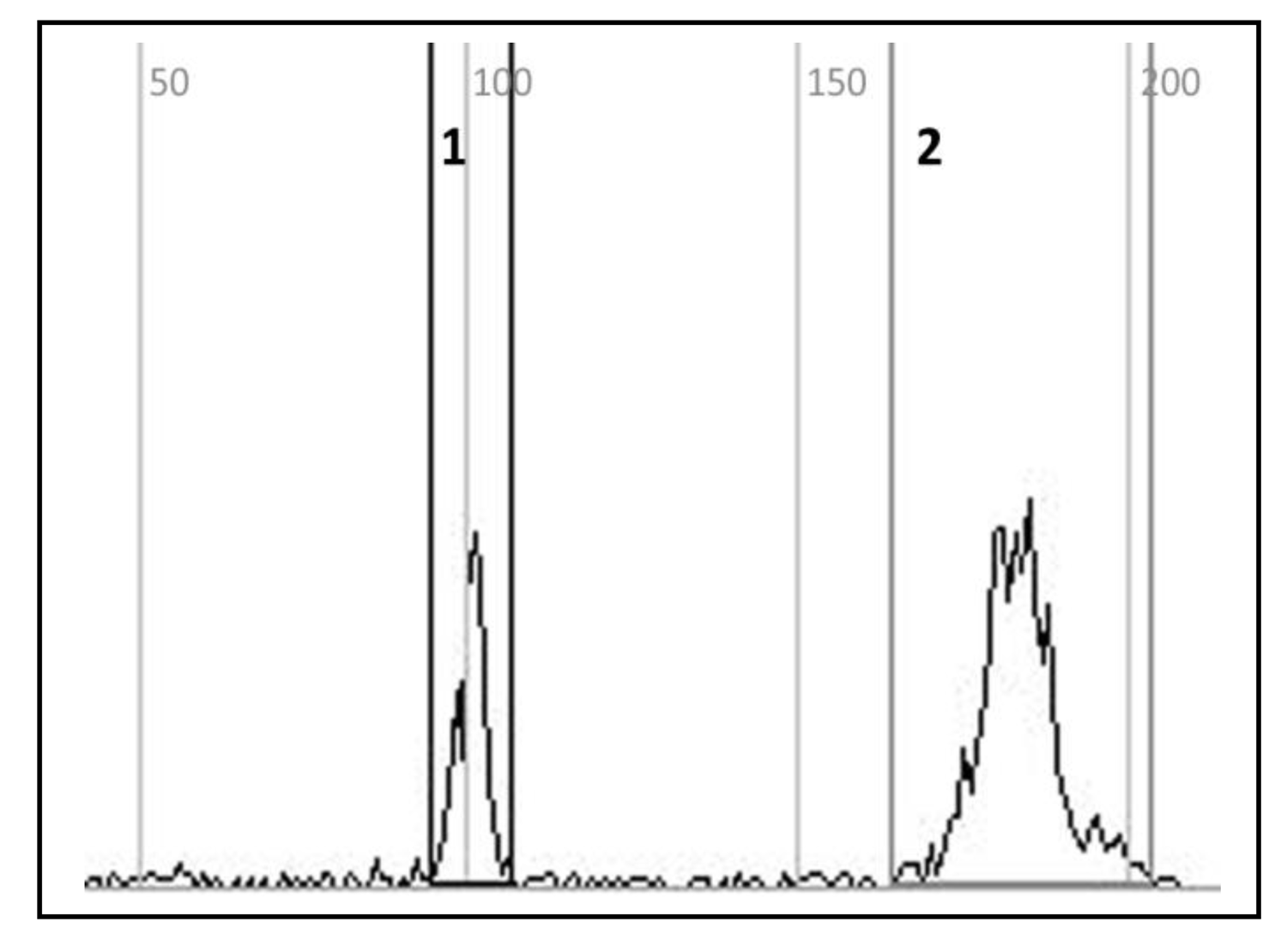

Supplement: S1 Fig — The tetraploid cultivar Trailhead (peak 1) and the octoploid cultivar Magnar (peak 2) were used as standards to distinguish ploidy in 110 wild collections from the intermountain Western USA. (TIF) [file pone.0148982.s002.tif]
