# Supplementary material for: Linking Genetic Variation in Adaptive Plant Traits to Climate in Tetraploid and Octoploid Basin Wildrye [Leymus cinereus (Scribn. & Merr.) A. Love] in the Western U.S
Source: PLoS One. 2016 Feb 16;11(2):e0148982. doi: 10.1371/journal.pone.0148982 (PMC4755535; doi:10.1371/journal.pone.0148982)
Supplement: S1 Table — Traits were measured in common gardens at Central Ferry and Pullman WA, in 2012 and 2013. (DOCX) [file pone.0148982.s003.docx]

| S1 Table. Pearson correlation coefficients between climate variables and plant traits for octoploid (n=57) and tetraploid (n=52) basin wildrye source locations. Traits were measured in common gardens at Central Ferry and Pullman WA, in 2012 and 2013. | | | | | | | | | | |
| --- | --- | --- | --- | --- | --- | --- | --- | --- | --- | --- |
| Trait | Climate variable^a^ | | | | | | | | | |
|  |  | MAT | TD | MAP | SHM | EMT | EXT | CMD | MAR | RH |
| **Phenology** |  |  |  |  |  |  |  |  |  |  |
| Heading | Octo | -0.01 | -0.47**^b^ | 0.39** | -0.20 | 0.34** | -0.40** | -0.53** | -0.32** | 0.49** |
|  | Tetra | -0.25 | -0.48** | 0.27* | -0.21 | 0.17 | -0.52** | -0.39** | 0.13 | 0.34* |
| Blooming | Octo | -0.02 | -0.48** | 0.28* | -0.13 | 0.30* | -0.36** | -0.42** | -0.23 | 0.46** |
|  | Tetra | -0.29* | -0.53** | 0.28* | -0.23 | 0.12 | -0.52** | -0.38** | 0.16 | 0.29* |
| Maturity | Octo | -0.06 | -0.39** | 0.15 | -0.11 | 0.18 | -0.32* | -0.30* | 0.01 | 0.29* |
|  | Tetra | -0.08 | -0.33** | 0.20 | -0.07 | 0.19 | -0.46** | -0.18 | 0.28* | 0.28* |
| **Morphology** |  |  |  |  |  |  |  |  |  |  |
| Leaf weight | Octo | 0.29* | -0.43** | 0.38** | -0.10 | 0.59** | -0.19 | -0.49** | -0.62** | 0.67** |
|  | Tetra | -0.22 | 0.22 | 0.17 | -0.31** | -0.32** | 0.11 | -0.17 | -0.43** | -0.14 |
| Leaf ratio | Octo | -0.28* | 0.48** | -0.40** | 0.13 | -0.59** | 0.23 | 0.48** | 0.52** | -0.66** |
|  | Tetra | 0.21 | -0.12 | -0.05 | 0.19 | 0.35** | -0.21 | 0.05 | 0.16 | 0.24 |
| Leaf area | Octo | 0.30* | -0.39** | 0.40** | -0.10 | 0.59** | -0.18 | -0.49** | -0.61** | 0.68** |
|  | Tetra | -0.27* | 0.22 | 0.16 | -0.33** | -0.40** | 0.14 | -0.17 | -0.43** | -0.20 |
| Specific leaf wt. | Octo | -0.02 | -0.24 | 0.04 | -0.01 | 0.08 | -0.09 | -0.03 | -0.08 | 0.06 |
|  | Tetra | 0.21 | 0.03 | 0.05 | -0.02 | 0.30* | -0.11 | -0.05 | -0.09 | 0.23 |
| Culm length | Octo | 0.29* | -0.09 | 0.06 | 0.17 | 0.33** | 0.09 | -0.06 | -0.31* | 0.29* |
|  | Tetra | -0.10 | 0.15 | -0.04 | 0.03 | -0.25 | 0.08 | 0.11 | -0.08 | -0.22 |
| Head length | Octo | 0.15 | -0.37** | 0.28* | -0.06 | 0.40** | -0.25 | -0.37** | -0.32** | 0.47** |
|  | Tetra | -0.32** | 0.17 | 0.13 | -0.29* | -0.36** | -0.02 | -0.22 | -0.32** | -0.09 |
| **Production** |  |  |  |  |  |  |  |  |  |  |
| Survival | Octo | 0.09 | 0.11 | 0.02 | -0.09 | 0.06 | 0.04 | -0.06 | -0.25 | 0.07* |
|  | Tetra | -0.09 | 0.23 | -0.10 | -0.15 | -0.38** | 0.16 | 0.11 | -0.09 | -0.35** |
| Head number | Octo | 0.18 | -0.34** | 0.32** | -0.16 | 0.44** | -0.24 | -0.43** | -0.51** | 0.55** |
|  | Tetra | -0.21 | 0.27* | 0.08 | -0.07 | -0.36** | 0.04 | 0.01 | -0.17 | -0.19 |
| Crown circum. | Octo | 0.28* | -0.35** | 0.40** | -0.16 | 0.56** | -0.22 | -0.52** | -0.63** | 0.68** |
|  | Tetra | -0.02 | 0.21 | -0.04 | -0.08 | -0.21 | 0.12 | 0.08 | -0.16 | -0.21 |
| ^a^MAT (mean annual temperature), TD (continentality, temp. difference between mean warmest and coldest months), MAP (mean annual precip.), EMT (30 year extreme min. temp.), SHM (summer heat-moisture index, (mean temp warmest month)/(mean summer precipitation/1000)), EXT (30 year extreme max. temp.), CMD, Hargreaves climatic moisture deficit (mm), MAR (mean annual solar radiation), RH (mean annual relative humidity).  ^b*,**^Significant at P<0.05 and P<0.01, respectively. | | | | | | | | | | |
