# Supplementary material for: Linking Genetic Variation in Adaptive Plant Traits to Climate in Tetraploid and Octoploid Basin Wildrye [Leymus cinereus (Scribn. & Merr.) A. Love] in the Western U.S
Source: PLoS One. 2016 Feb 16;11(2):e0148982. doi: 10.1371/journal.pone.0148982 (PMC4755535; doi:10.1371/journal.pone.0148982)
Supplement: S2 Table — (DOCX) [file pone.0148982.s004.docx]

| S2 Table. Canonical correlation summary for the first four variates relating plant traits from common gardens and source climates for basin wildrye. | | | | |
| --- | --- | --- | --- | --- |
| Statistic | Canonical variate | | | |
|  | 1 | 2 | 3 | 4 |
| Canonical correlation | 0.82 | 0.69 | 0.59 | 0.49 |
| Proportion of variation | 0.49 | 0.21 | 0.13 | 0.08 |
| Cumulative proportion | 0.49 | 0.70 | 0.83 | 0.91 |
| F-value | 2.87 | 2.01 | 1.53 | 1.16 |
| P-value^*^ | <0.0001 | <0.0001 | 0.0057 | 0.2178 |
| ^*^Likelihood approximations testing if a canonical variate and all that follow are equal to zero. | | | | |
