# Supplementary material for: Linking Genetic Variation in Adaptive Plant Traits to Climate in Tetraploid and Octoploid Basin Wildrye [Leymus cinereus (Scribn. & Merr.) A. Love] in the Western U.S
Source: PLoS One. 2016 Feb 16;11(2):e0148982. doi: 10.1371/journal.pone.0148982 (PMC4755535; doi:10.1371/journal.pone.0148982)
Supplement: S3 Table — (DOCX) [file pone.0148982.s005.docx]

| S3 Table. Regression models between traits and climates for octoploid (n=57) and tetraploid (n=52) source populations of basin wildrye in the intermountain West, U.S.A. | | |
| --- | --- | --- |
| Trait | R-square | Model^a^ |
| Heading day | 0.39 | 279.249 - 2.89866 (MAT) + 0.9758 (TD) + 1.86561 (EMT) - 0.78642 (EXT) - 0.61439 (RH) |
| Leaf area cm^2^ | 0.47 | 324.251 - 5.40921 (MAT) + 4.05382 (TD) + 5.41222 (EMT) - 0.05243 (CMD) - 2.58705 (RH) |
| Head number | 0.29 | 971.068 - 30.3231 (MAT) + 15.21722 (TD) + 0.03947 (MAP) + 19.50437 (EMT) - 6.68335 (RH) |
| Canonical variate 1 | 0.64 | 44.6960 + 0.34901 (TD) - 0.00274 (MAP) + 0.50965 (EMT) - 0.22709 (EXT) - 0.01292 (CMD) - 0.32756 (RH) |
| Canonical variate 2 | 0.38 | 17.9873 - 1.46495 (MAT) + 0.43951 (TD) + 0.00567 (MAP) + 0.66059 (EMT) + 0.01357 (CMD) - 0.13338 (RH) |
| ^a^MAT (mean annual temperature), TD (continentality, temp. difference between mean warmest and coldest months), MAP (mean annual precip.), EMT (30 year extreme min. temp.), EXT (30 year extreme max. temp.), CMD (Hargreaves climatic moisture deficit), RH (mean annual relative humidity). | | |
